# Organic Mixed Ionic–Electronic Conductors for Bioelectronic Sensors: Materials and Operation Mechanisms

**DOI:** 10.1002/advs.202306191

**Published:** 2023-12-26

**Authors:** Hyunwook Kim, Yousang Won, Hyun Woo Song, Yejin Kwon, Minsang Jun, Joon Hak Oh

**Affiliations:** ^1^ School of Chemical and Biological Engineering Institute of Chemical Processes Seoul National University 1 Gwanak‐ro Gwanak‐gu Seoul 08826 Republic of Korea

**Keywords:** neural interfacing, neuromorphic devices, organic bioelectronics, organic mixed ionic**–**electronic conductor (OMIEC), sensors

## Abstract

The field of organic mixed ionic‐electronic conductors (OMIECs) has gained significant attention due to their ability to transport both electrons and ions, making them promising candidates for various applications. Initially focused on inorganic materials, the exploration of mixed conduction has expanded to organic materials, especially polymers, owing to their advantages such as solution processability, flexibility, and property tunability. OMIECs, particularly in the form of polymers, possess both electronic and ionic transport functionalities. This review provides an overview of OMIECs in various aspects covering mechanisms of charge transport including electronic transport, ionic transport, and ionic–electronic coupling, as well as conducting/semiconducting conjugated polymers and their applications in organic bioelectronics, including (multi)sensors, neuromorphic devices, and electrochromic devices. OMIECs show promise in organic bioelectronics due to their compatibility with biological systems and the ability to modulate electronic conduction and ionic transport, resembling the principles of biological systems. Organic electrochemical transistors (OECTs) based on OMIECs offer significant potential for bioelectronic applications, responding to external stimuli through modulation of ionic transport. An in‐depth review of recent research achievements in organic bioelectronic applications using OMIECs, categorized based on physical and chemical stimuli as well as neuromorphic devices and circuit applications, is presented.

## Graphical abstract

The recent development of organic bioelectronics based on organic mixed ionic**–**electronic semiconductors/conductors (OMIECs) is reviewed. Multifunctional properties of OMIECs including sensing, energy harvesting/storage, information transfer, and actuation enable them to function as the active component of multiple devices such as transistors, sensors, electrochemical transistors, electro/photochromic devices, and artificial synapses.

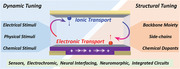

## 1 Introduction

During the late 1990s and early 2000s, there was increasing interest in the concept of mixed ionic electronic conductors, which possess the ability to conduct both electrons and ions. At that time, most research efforts were directed toward inorganic materials, particularly perovskite oxides.^[^ ^1^, ^2^, ^3^ ^]^ These materials composed of transition metal oxides, demonstrated encouraging mixed conductivity characteristics. When organic materials showed promise in the area of conductors and semiconductors—with several advantages compared to inorganic materials, such as solution processability, flexibility, and tunable properties—researchers expanded their study of mixed conduction beyond inorganic materials to include organic materials, especially polymers.^[^ ^4^, ^5^ ^]^ This led to the investigation of a wide range of organic substances, such as conjugated polymers, small molecules, and organic‐inorganic hybrids.

Studies on organic conductors/semiconductors have traditionally focused on electronic transport, particularly in the context of π‐conjugated polymers. However, in recent years, there has been a significant increase in attention toward the necessity of ionic transport and the combined influence of ionic and electronic properties, due to their unique electrical characteristics. Organic materials that effectively facilitate both ionic and electronic transport have emerged as promising candidates for various applications, ranging from energy storage devices to bioelectronics.^[^ ^6^, ^7^, ^8^, ^9^, ^10^, ^11^, ^12^, ^13^, ^14^, ^15^ ^]^ These materials, known as organic mixed ionic–electronic conductors (OMIECs), are highly favorable due to their processability, excellent performance, and the potential for improved charge storage and coupled transport properties. OMIECs are often in the form of polymers that have electronic and ionic transporting parts. For example, the most well‐known OMIEC, poly(3,4‐ethylenedioxythiophene):poly(styrenesulfonate) (PEDOT:PSS), has electronic conductive PEDOT—due to the electron conducting nature of the polythiophene backbone—and insulating PSS. PEDOT:PSS enables ionic transport by the polyelectrolyte, PSS, as well as polarized charges between PEDOT (positive charge) and PSS (negative charge).^[^ ^16^ ^]^

As interest in applying OMIECs to various devices has grown, numerous types of new OMIECs have been developed.^[^ ^16^, ^17^ ^]^ However, the way forward is still long and complicated. To develop OMIECs for multiple applications and a variety of purposes, research efforts have focused on the mechanisms of ionic and electronic transport, as well as ionic‐electronic coupling.^[^ ^18^ ^]^ A deep comprehension of the interconnections between ionic transport, electronic transport, and ionic–electronic coupling is essential to the field of OMIECs. Despite numerous investigations into ionic transport in polyelectrolytes and solid polymer electrolytes, as well as electronic charge transport in conjugated organic materials, the unique ionic–electronic coupling in OMIECs has yet to be fully revealed. Thus far, studies have clarified that this property depends on diverse factors such as processing conditions, chemical structure, morphology, and electrolyte selection.^[^ ^18^ ^]^

OMIECs have been utilized in a wide range of areas including batteries,^[^ ^19^, ^20^ ^]^ supercapacitors,^[^ ^21^ ^]^ actuators,^[^ ^22^ ^]^ light‐emitting electrochemical cells,^[^ ^23^ ^]^ chemical sensors,^[^ ^8^, ^24^ ^]^ bioelectronics,^[^ ^9^ ^]^ ion pumps,^[^ ^25^ ^]^ organic electrochemical transistors (OECTs) and OECT‐based neuromorphic and memory devices as active layers,^[^ ^26^ ^]^ polymer electrodes and so on. The strength of the ionic–electronic coupling in OMIECs plays a critical role in determining how much energy OMIEC‐based batteries and capacitors can store. Simultaneously, the charging rates and power capability are often restricted to the rate of ionic transport.^[^ ^18^ ^]^ Furthermore, ionic transport matters in transient behaviors and is essential for the operation of devices such as light‐emitting electrochemical cells.^[^ ^12^, ^18^, ^27^, ^28^ ^]^

For organic bioelectronics in particular, including neuromorphic devices, electrochromic devices, and sensors that respond to several types of external stimuli, OMIECs have great potential due to their unique properties such as biodegradability and compatibility with biological systems. Compatibility with both biological systems and their operating mechanisms are important factors for OMIECs to be promising candidates for the active layers of bioelectronics. Biological systems rely on the modulation of electronic conduction and ionic transport to facilitate physiological and neural activities. The operation principles of OMIECs closely resemble those of such systems, and OMIEC‐based electronics, such as resistors and OECTs, also have a promising possibility to imitate the systems.^[^ ^29^, ^30^, ^31^, ^32^, ^33^ ^]^ Through the application of an external stimulus—for example, a gate voltage, which controls the modulation of ionic transport in the electrolyte—the charge carrier concentration in the active layer undergoes changes.^[^ ^34^ ^]^ As a result, electronic devices based on OECTs hold significant appeal for a wide range of bioelectronic applications.

This review provides an overview of conducting/semiconducting conjugated polymer‐based OMIEC materials and their applications in organic bioelectronics such as numerous and various (multi)sensors, neuromorphic devices, and electrochromic devices. This review is structured as follows. In chapter 2, we briefly discuss the current understanding of the charge transport physics of OMIEC. Chapter 3 presents a brief review of recent conducting/semiconducting conjugated polymers used as OMIECs. In chapter 4, we review recent research achievements concerning organic bioelectronic applications using OMIECs. We subdivide this chapter according to 1) chemical/biosensor, 2) pressure sensor, 3) neural interfacing, 4) neuromorphic devices and circuit applications, and 5) electrochromic devices. Finally, this review concludes with an outlook on how these advances in materials will open up new scientific opportunities to address the performance requirements of next‐generation applications.

## 2 Basic Physical Processes in OMIECs

While our understanding of electronic transport in OMIECs relies on studies involving molecular and electrochemically doped conjugated polymers, that of ionic transport is dependent on studies focused on electrically insulating polymer electrolytes and polyelectrolytes. Detailed descriptions of the mechanisms of ionic and electronic transport across various conditions can be found in the scientific literature.^[^ ^35^ ^]^ In this chapter, therefore, we focus on discussing the fundamental physical processes of electronic and ionic transport in OMIECs as well as the processes in OECTs.

### 2.1 Electronic Transport in OMIECs

**Figure 1:**
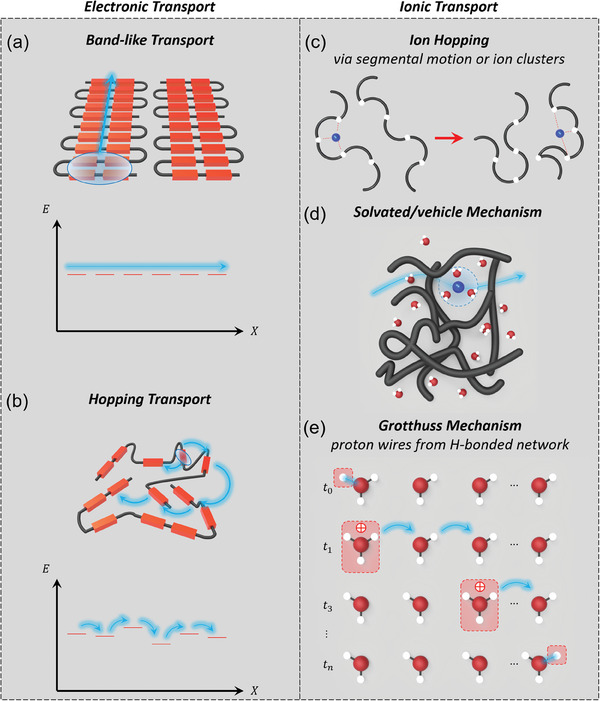
Schematic illustration of charge transport. Electronic charge transport mechanisms: molecular and energy schematic of a) band‐like transport of a relatively delocalized electronic charge carrier and b) thermally activated hopping transport of a relatively localized electronic charge carrier. Ionic charge transport mechanisms: c) cation hopping assisted by segmental motion or ion cluster, d) solvated ion vehicle transport, and e) the Grotthus mechanism of proton hopping. In the left column, a polymer part in which electronic charge transport takes place is shown in the red block and black lines indicate a polymer chain. In the right column, blue, red, and white circles exhibit cations, O atoms, and H atoms, respectively. Polymers are shown in black lines. Both electronic and ionic charges are shown in cyan.

Conjugated polymers weakly bonded by van der Waals force, resulting in many degrees of conformational freedom and weakly interacting with each other, provide a unique electronic conduction system in which transport is intermediate between conventional hopping transport (low mobility) in the amorphous phase and band transport (high mobility) in the covalently bonded ordered phase (**Figure** **1**a,b).^[^ ^36^, ^37^, ^38^, ^39^ ^]^ Generally, OMIECs often exhibit a significant degree of structural disorder because most of them consist of conjugated polymers. In the high degree of π‐conjugation, there is a continuous pathway of delocalized π‐orbitals. This allows weakly bound electrons to migrate more easily along an adjacent molecule and between molecules. In contrast, the high degree of structural disorder leads to a low degree of delocalization and insufficient π‐π overlapping, resulting in thermally activated hopping transport and the subsequent decline in electronic transport characteristics.

The chemical structure of a repeating unit of the polymer has a great impact on electronic charge transport. By increasing the length of the side chain from poly(3‐hexylthiophene‐2,5‐diyl) (P3HT) to poly(3‐octylthiophene‐2,5‐diyl), the electronic charge mobility is reduced from 1.1 × 10^−2^ to 1.4 × 10^−4^ cm^2^ V^−1^ s^−1^.^[^ ^40^, ^41^ ^]^ With branching side chains, a similar phenomenon is observed; applying an ethyl‐branch into a hexyl side chain, poly(3‐(2‐ethylhexyl)thiophene), decreases the field‐effect mobility by an order of magnitude.^[^ ^42^ ^]^ By contrast, there are many strategies for boosting electronic charge transport; enhancing the rigidity of the polymer backbone,^[^ ^43^ ^]^ synthesizing donor‐acceptor copolymers,^[^ ^44^, ^45^, ^46^, ^47^, ^48^, ^49^ ^]^ adjusting surface energy levels,^[^ ^50^, ^51^ ^]^ and increasing regioregularity^[^ ^52^, ^53^ ^]^ can improve electronic charge transport. However, the aforementioned strategies would be expected to contribute negatively to ionic conduction.^[^ ^54^ ^]^

Without doping, both the density of accessible states by hopping transport and the electronic charge carrier density are very low. Therefore, electronic charge carrier mobility and electrical conductivity are also low. In OMIECs, however, doping thanks to ionic–electronic coupling also exists. At very low doping concentrations, doping only fills charge trap states in the organic semiconductor; no free electronic charge carriers are generated and the dopant ions after the doping process serve as trap sites for the electronic charge carrier.^[^ ^55^, ^56^, ^57^ ^]^ Once the doping concentration exceeds the charge trap density, the activation energy for hopping transport decreases, and electronic charge carrier mobility increases. At high doping concentrations, some OMIECs show band transport, and high electronic charge carrier mobility is observed. However, at extremely high doping concentrations, a reduction in crystallinity is observed, leading to a decrease in electronic charge carrier mobility in most cases.^[^ ^58^, ^59^ ^]^ Scientific reviews providing detailed explanations and descriptions of charge transport in organic semiconductors are abundant, so here we will highlight the basic concepts.^[^ ^60^, ^61^, ^62^, ^63^ ^]^

### 2.2 Ionic Transport in OMIECs

As OMIEC can serve as the ionic pathway, the capability of ionic transport in the polymer electrolyte is one of the main characteristics to determine the performance of bioelectronic devices based on OMIECs.^[^ ^64^ ^]^ In the dry state of OMIECs, the polymer itself is macroscopically immobile. ion migration takes place through ion hopping, which is facilitated by the segmental motion of the side chains or the backbone chain (short polymer segments) in the vicinity of the ion (Figure 1c). The ion hopping mechanism is dependent on the temperature and then ion conductivity could be described by following the equation below the glass transition temperature (*T*_g_)^[^ ^65^, ^66^, ^67^, ^68^ ^]^:

(1) $$\sigma =\sigma_{0}\exp\left(\frac{-E_{a}}{k_{B}T}\right)$$

where σ_0_ is a pre‐exponential factor. *E*_a_ and *k*_B_ indicate the activation energy and Boltzmann constant, respectively. *T* is the temperature. However, at higher temperatures than the glass transition temperature of polymer electrolyte‐based OMIECs, the ionic conductivity, which is dependent on the temperature, begins not to follow the Arrhenius behavior because ionic transport is dominantly under control of the effective viscosity of the OMIECs rather than a hopping barrier. When the viscosity of the OMIEC decreases with temperature, the Vogel–Tammann–Fulcher (VTF) relation (Equation 2) is more suitable for describing ion conductivity.^[^ ^64^ ^]^

(2) $$\sigma =\sigma_{0}\;T^{-1/2}\exp\left(\frac{-E_{a}}{k_{B}\left(T-T_{0}\right)}\right)$$

where σ_0_ is a pre‐exponential factor, *E*_a_ is also an activation energy related to segmental motion. *T*_0_ is referred to as a reference temperature or the Vogel temperature, equal to the glass transition temperature in ideal glasses.^[^ ^69^, ^70^ ^]^ *T* is a given temperature. As described above, ionic transport from an occupied site to a vacant site is thermally activated and is strongly influenced by the chain flexibility of OMIECs. However, adding additives such as molecules of propylene carbonate (PC) into OMIECs can make OMIECs more flexible and enhance the segmental motion through the plasticizing effect. Furthermore, in the swollen state of OMIECs, small polar solvents can enhance ionic transport by solvating either ionic layers of OMIECs or mobile ions. The interaction between the mobile ions and the ionic layer of polymer electrolyte‐based OMIECs is weakened as molecular solvents contribute to ion coordination (Figure 1d).^[^ ^71^, ^72^ ^]^ In addition, how ions are transported can be heavily affected by what type of ion moves. For example, protons (H^+^) are well known to diffuse fast through the Grotthuss mechanism between water molecules or hydrogen‐bonded networks that involve the exchange of a covalent bond.^[^ ^73^, ^74^, ^75^ ^]^

The ability to transport ions and hence conduct ionic currents (in addition to electronic currents) by ion hopping (via segmental motion or ion clusters), solvated/vehicle, and Grotthuss mechanisms sets OMIECs apart from other π‐conjugated organic semiconductors (Figure 1c–e).

The negatively (or positively) charged ions can be considered analogous to electrons (or holes). Compared to electronic transport, however, ionic transport is enhanced by a lesser‐density polymer structure due to accommodating the relatively large size of ions.^[^ ^76^ ^]^ In addition, ionic transport is much more complex because of the nature of ions; ions can be multi‐valent, and present in multiple species, and the larger the ion, the slower the rate of ion migration through the polymer structure and, hence, the lower the ionic transport.^[^ ^77^, ^78^, ^79^ ^]^ Furthermore, ions are easily influenced by which solvent is used and the solvation process. Ionic transport can be quantified by the parameter called ionic conductivity (σ_ionic_), which is the sum of the individual ion conductivity for each mobile ionic species, denoted as i; the sum of number density (*n*_i_), the products of the ion charge (|*z*_i_|), elementary charge (*e*), and ion mobility (*µ*_i_):

(3) $$\sigma_{\mathrm{ionic}}=\sum_{i}n_{i}\;\left|z_{i}\right|e\mu_{i}$$

where *n*_i_ is the sum of number density, |*z*_i_| represents the products of the ion charge, *e* is the elementary charge, and *µ*_i_ is ion mobility.

Here, ion mobilities are convertible to diffusion coefficients (*D*) via the Einstein–Smoluchowski relation (Equation 4) or vice versa, where *k*_B_ is Boltzmann's constant and *T* is temperature:

(4) $$D=\frac{\mu k_{B}T}{e}$$

To enhance ion conductivity, it is necessary to incorporate components that can facilitate ionic transport. Simply relying on the backbone of the conjugated polymer alone may not effectively drive mobile ions. Therefore, improvements have been made by making a blend of a conjugated polymer with either a polyelectrolyte or a polymer electrolyte (heterogeneous blends),^[^ ^8^, ^80^, ^81^, ^82^ ^]^ or by synthesizing a new block co‐polymer that combines a conjugated polymer with either a polyelectrolyte or a polymer electrolyte (heterogeneous block co‐polymers).^[^ ^83^, ^84^, ^85^ ^]^ OMIECs fabricated using these approaches exhibit heterogeneous morphologies characterized by the presence of predominately electron‐conducting and ion‐conducting domains that are microphase segregated. However, to make homogeneous OMIECs with no microphase segregation between electron‐conducting and ion‐conducting domains, a new concept of conjugated polymers was synthesized with polar side chains (conjugated polymer electrolytes)^[^ ^86^, ^87^, ^88^ ^]^ or side chains charged with pendant ions (conjugated polyelectrolytes).^[^ ^6^, ^89^, ^90^ ^]^

### 2.3 Ionic–Electronic Coupling in OMIECs

Ionic–electronic coupling plays a fundamental role in the performance and functionality of OMIECs. There are still various to explain the ionic–electronic coupling in OMIECs and they are open to dispute. There are two main explanations.^[^ ^18^ ^]^ One is the charge coupling/stabilization through electrostatic interactions between cations (or anions) and electrons in *n*‐type (or holes in *p*‐type) OMIECs (compensation doping). The volumetric nature of this coupling results from the ability of the electrolyte to permeate the entire volume of the OMIEC film, extending beyond the film surface or film/electrolyte interface. At the molecular level, ions can be thought to stabilize nearby electronic charges on the conjugated polymer backbone, such as poly(thiophene), based on electrostatic interaction. The other thing is direct charge transfer observed in some OMIECs including protonation of polyaniline or polypyrrole.^[^ ^18^, ^91^, ^92^ ^]^ The degree of ionic–electronic coupling is sensitive to the application of a potential. This results in a potential–dependent capacitance (*C*), which characterizes the strength of the ionic–electronic coupling. Homogeneous OMIECs exhibit enhanced ionic–electronic coupling and higher volumetric capacitances compared to their heterogeneous counterparts.^[^ ^18^ ^]^

To fully comprehend and utilize the potential of ionic–electronic coupling in OMIECs, it is essential to understand fully the interaction between ionic and electronic transport as well as the dependence on processing conditions, chemical structure, morphology, and electrolyte selection. Finally, establishing a robust understanding of the fundamental material structure–property relationship is also important.

### 2.4 Operation in OECTs

Many organic bioelectronic applications that aim to interface with biology mimic biological signaling systems and hence implement biological sensing, physiological signal recording, and neuromorphic processing, have counted on OECTs, which have the advantages of highly efficient signal transduction and amplification.^[^ ^34^ ^]^ Biological systems, including physiological and neural activities, are based on the modulation of electronic conduction and ionic transport. The operation principles of OECTs are very similar to this system; by applying the gate voltage (*V*_G_), which induces the modulation of ionic flux in the electrolyte, changes in the charge carrier concentration in the active layer occur. Therefore, OECT‐based electronic devices are particularly attractive for various bioelectronic applications.

The amplification property of OECTs is assessed using the transconductance (*g*_m_), which can be determined by the following equation.

(5) $$g_{m}=\frac{\partial I_{D}}{\partial V_{G}}=\frac{Wd}{L}\;\mu C^{*}\left(V_{\mathrm{th}}-V_{G}\right)$$

where, *L*, *W*, and *d* are the channel length, width, and thickness, respectively, µ is charge carrier mobility, and *V*_th_ is the threshold voltage of the active layer. C* is volumetric capacitance and represents the extent of ion storage, penetration, and transport ability of OMIECs, while µ reflects the ability to transport electronic charges across the channel. Therefore, the figure of merit of the channel material is defined by the product of these two parameters. Organic field‐effect transistors (OFETs) accumulate mobile electronic charges (holes for *p*‐type and electrons for *n*‐type organic semiconductors) near the interfaces with the dielectric layer by applying a gate bias, utilizing the field effect.^[^ ^93^ ^]^ Conversely, OECTs regulate the conductivity of the channel layer through ion injection/extraction. Since this process occurs throughout the entire volume of the active layer, the capacitance should take into account the full volume of the active layer.^[^ ^94^ ^]^ According to Equation (5), the high performance of OECTs can be achieved by optimizing either the device geometry or the µC* product of OMIECs.

**Figure 2:**
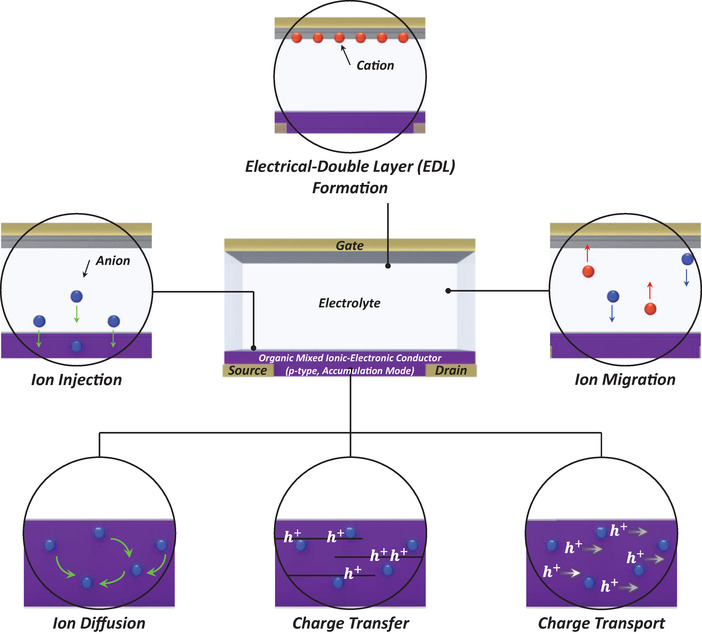
Basic physical processes in OECTs. Generally, OECT operation based on *p*‐type OMIEC in accumulation mode consists of the following six processes: 1) ion migration in the electrolyte induced by the gate bias voltage, 2) electrical double layer formation at the gate electrode/electrolyte and electrolyte/channel interfaces, 3) ion injection across the electrolyte/channel interface, 4) ion diffusion in the channel layer, 5) charge transfer (electrochemical oxidation/reduction), and 6) charge carrier transport in the channel layer. In 5) and 6), *h* ^+^ represents charge carriers (holes).

Conventional OECTs have two operation modes: accumulation and depletion. The difference between these two modes originates from the intrinsic doping state of the active layer. If the active layer is in a doped state and made of a conducting polymer, the device should operate in depletion mode (from an ON to an OFF state). In contrast, a semiconducting polymer with very low electronic conductivity should work in accumulation mode (from an OFF to an ON state).^[^ ^94^ ^]^ Representative materials used as an active layer of OECTs operating in the accumulation mode are conjugated polymers.^[^ ^95^ ^]^ For the following discussion, the OECT is assumed to have a *p*‐type channel and operate in accumulation mode, where the majority of charge carriers are holes. A typical OECT can be divided into six physical phenomena that govern charge transport, as illustrated in **Figure** **2**: 1) ion migration in the electrolyte induced by a gate voltage, 2) formation of an electrical double layer at both the gate electrode/electrolyte and electrolyte/channel interfaces, 3) ion injection across the electrolyte/channel interface, 4) ion diffusion in the active layer, 5) electrochemical charge transfer in the active layer, and 6) charge carrier transport in the active layer. In other words, when a gate voltage is applied, it causes mobile ions in the electrolyte to accumulate at the electrolyte/channel interface and even penetrate the active layer. This accumulation and penetration of the ions alters the doping level of the organic semiconductor, which in turn modulates the electronic conduction throughout the channel. As a result, the output signals of OECTs are significantly influenced by the interactions between electrons and ions. For more comprehensive and detailed discussions on OECT operation, we recommend referring to previous literature reviews.^[^ ^96^ ^]^

The time it takes for OECTs to switch ON or OFF is longer than OFETs because OECTs operation includes ion migration and penetration inside the channel layer.^[^ ^94^, ^95^ ^]^ Unlike OFETs, which typically operate up to the MHz range, OECTs generally function in ≈100 kHz range.^[^ ^88^, ^97^, ^98^, ^99^ ^]^ The Bernards model (Equation 6) predicts that the response time is limited by either the electronic circuit, which is described using Ohm's law, or the ionic circuit, which is modeled by a resistor (*R*_S_) and a capacitor (*C*_d_) in series.^[^ ^11^, ^100^ ^]^

(6) $$I_{D}\;\left(t\right)=I_{\mathrm{SS}}\;\left(V_{G}\right)+\Delta I_{\mathrm{SS}}\left[1-f\frac{\tau_{e}}{\tau_{\mathrm{ON}}}\right]\exp\left(-\frac{t}{\tau_{\mathrm{ON}}}\right)$$

where *I*_SS_ and Δ*I*_SS_ are the steady–state drain current under the fixed *V*_G_ and the difference between the initial (an ON state) and final (an OFF state) steady‐state drain currents, respectively. *f* represents a weighting factor that corresponds to the contribution of *I*_G_ to *I*_D_. τ_e_ and τ_ON_ are equivalent to the electronic transit time (τ_e_ ≡ *L* ^2^/µ*V*_D_) in the channel and the ionic RC time constant (τ_ON_ ≡ *R*_S_ *C*_CH_), respectively. The capacitance of the channel, *C*_CH_, is proportional to the channel volume, while the resistance of the electrolyte, *R*_S_, scales linearly in relation to $1/\sqrt{WL}$.^[^ ^101^ ^]^ According to Equation (6), when the speed of the electronic transport exceeds that of the ionic transport, the drain current, *I*_D_, experiences a mono‐decay exponential decrease until it reaches its steady‐state current level (monotonous relaxation behavior). On the other hand, when the ionic transport is faster than the electronic transport, *I*_D_ initially spikes over its original level and then exponentially recovers to its final current level (spike and recovery behavior). These two types of behavior have been observed in various OECTs.^[^ ^102^, ^103^, ^104^, ^105^ ^]^

### 2.5 Characterization of Properties of OMIECs

As previously described, OMIECs represent a complex and complicated case involving multiple charged species that interact intensively. This adds a layer of complexity to the understanding and electrical and electrochemical characterization of these materials. Therefore, the accurate isolation and quantification of ionic and electronic transport in OMIECs is a significant challenge due to the complex interaction of these charged species. Electrochemical impedance spectroscopy (EIS) can be a useful tool for separating and understanding how ions and electrons move at the same time, as well as for measuring how much they interact with each other.^[^ ^106^, ^107^, ^108^ ^]^ EIS modifies the frequency of the applied potential in order to extract the real and imaginary components of the complex impedance, which gives us information on the OMIEC's ability to conduct and store charge, respectively. EIS has also been used to determine the mobility and conductivity of electronic and ionic transport, as well as to quantify ionic–electronic coupling, which can be represented as volumetric capacitance or electrochemical density of states.^[^ ^109^, ^110^, ^111^, ^112^ ^]^

OECT could be a promising candidate for isolating and characterizing electronic charge transport of the OMIEC which is dependent on the ionic–electronic coupling. In conventional OECTs, an OMIEC thin film serves as the channel, while the gate dielectric is replaced with an ion‐conducting electrolyte. The structure of OECTs allows for the separation of electronic currents – which flow laterally within the channel between the source and drain electrode – from the ionic charging currents that exist within the electrolyte between the gate electrode and the channel.^[^ ^34^ ^]^ That is, the source‐drain current reflects electronic transport, while the gate current is able to indicate ionic transport. The gate voltage adjusts the 3D ionic–electronic coupling (or doping) within the OMIEC channel, thereby altering the electrical conductivity. The electronic charge carrier mobility can be determined by estimating the electronic charge carrier density from the volumetric capacitance identified by EIS or by integrating the gate currents.^[^ ^59^ ^]^

## 3 Materials

### 3.1 Organic Conductors

#### 3.1.1 PEDOT:PSS

**Figure 3:**
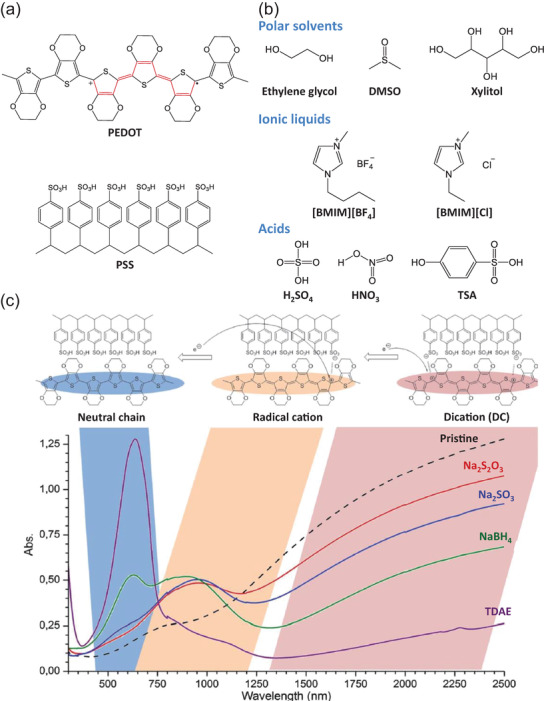
a) Chemical structures of PEDOT and PSS. b) Chemical structures of various dopants for PEDOT:PSS. c) UV–vis–NIR spectra of PEDOT chains treated with various reducing agents. Reproduced with permission,^[^ ^126^ ^]^ Copyright 2014, Royal Society of Chemistry.

PEDOT:PSS, one of the most commonly used OMIECs, is a blended system consisting of conductive PEDOT and insulating PSS (**Figure** **3**a). In 1988, PEDOT was first obtained by researchers from Bayer using an oxidation of 3,4‐ethylenedioxythiophene (EDOT) and its thin film showed high stability and conductivity.^[^ ^113^ ^]^ Despite such desirable properties, it was not feasible to coat PEDOT onto a substrate because it could not be redissolved or dispersed once it was polymerized. To overcome the solubility issue, Bayer's scientists polymerized EDOT in water in the presence of PSS, which is a polyelectrolyte template. Due to the Columbic interaction between positive charges in PEDOT and negative charges in PSS, the aqueous micro‐dispersion of PEDOT stabilized with PSS^−^ became solution processable with high stability, which is suitable for academic and industrial applications.^[^ ^114^ ^]^

Pristine PEDOT:PSS has higher electrical conductivity (0.1–1 S cm^−1^) than PEDOT alone due to the positive doping from PSS^−^.^[^ ^115^, ^116^ ^]^ However, to be utilized in various bioelectronic applications, the conductivity of PEDOT:PSS should be tunable. Researchers have tuned the electrical conductivity of PEDOT:PSS film through secondary doping (e.g., treatment with polar solvents,^[^ ^117^, ^118^, ^119^ ^]^ ionic liquids,^[^ ^120^ ^]^ and acids^[^ ^121^, ^122^ ^]^), and the electronic conductivity reached a maximum value > 4000 S cm^−1^ (Figure 3b).^[^ ^123^ ^]^ In addition, PEDOT:PSS shows ionic conductivity (≈1 S cm^−1^) due to the PSS polyelectrolyte.^[^ ^116^ ^]^ The ionic transport in PEDOT:PSS is conducted in the PSS‐rich region, but it is difficult to distinguish between ionic and electronic transport regions due to the considerable ionic–electronic coupling.^[^ ^18^ ^]^

Another fascinating property of PEDOT:PSS is its tunable optical transparency, which is up to 90% for the visible range.^[^ ^124^ ^]^ The potential of the optically transparent PEDOT:PSS film is huge in bioelectronics, such as neural interfaces, e‐skins, and human‐interactive optical devices.^[^ ^125^, ^126^ ^]^ Furthermore, the optical property can be tuned by varying the doping level. Neutral PEDOT shows a purple/blue color and has an optical absorption gap of ≈1.5 eV. As shown in Figure 3c, when PEDOT is oxidized, new energy states are introduced in the bandgap in the near‐infrared region outside the visible range, and PEDOT becomes transparent in the visible range.^[^ ^123^ ^]^ This feature allows PEDOT:PSS to be used in various applications including electrochromic fields.

#### 3.1.2 PEDOT Derivatives

**Scheme 1:**
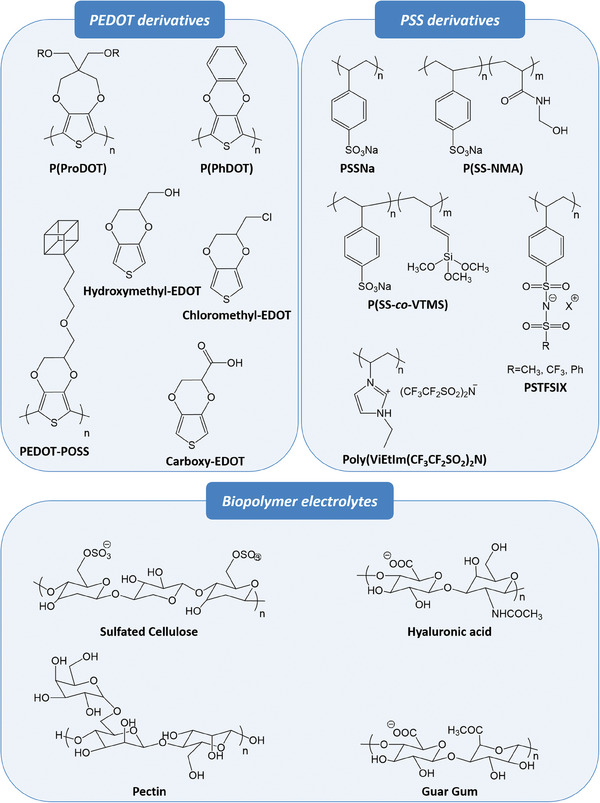
Chemical structures of PEDOT, PSS derivatives, and biopolymer electrolytes.

Despite the desirable properties mentioned above and its commercial availability, PEDOT:PSS has many limitations including the high acidity and hygroscopicity of PSS and low bio‐functionality.^[^ ^114^, ^116^, ^127^, ^128^, ^129^ ^]^ In terms of bioelectronics, the high acidity of PPS can undermine the stability of electronic devices and be incompatible with the human body. The acidity and hygroscopicity were handled by introducing a hydrophobic group into PSS (**Scheme** **1**). For example, sodium 4‐styrenesulfonate (SSNa), N‐(methylolacrylamide) (NMA),^[^ ^130^ ^]^ and vinyltrimethoxysilane (VTMS)^[^ ^131^ ^]^ were used as comonomers and the resulting PSS‐Na, P(SS‐NMA), and P(SS‐co‐VTMS) copolymers showed improved hydrophobicity and thermal stability. In addition, new polyelectrolytes, including poly(1‐vinyl‐3‐ethylimidazolium bispentafluoromethanesulfonimide) (poly(ViEtIm(CF_3_CF_2_SO_2_)_2_N),^[^ ^132^ ^]^ lignosulfonates (SLs),^[^ ^133^ ^]^ methylnaphthalene sulfonate formaldehyde condensates (MNSFs)^[^ ^134^ ^]^ and poly(potassium 4‐styrenesulfonyl (trifluoromethylsulfonyl) imide) (PSTFSIK)^[^ ^135^ ^]^ were developed as alternatives to PSS to reduce the acidity of PEDOT film. Studies on EDOT‐derivatives that are soluble in water or organic solvents alone have also been conducted, such as alkoxy‐functionalized 3,4‐propylenedioxythiophenes (ProDOT),^[^ ^136^ ^]^ 3,4‐phenylenedioxythiophenes (PheDOTs),^[^ ^137^ ^]^ and PEDOT‐POSS.^[^ ^138^ ^]^ These derivatives were free from the high acidity and hygroscopicity originated from polyelectrolytes.

In addition to acidity and hygroscopicity, the low bio‐functionality of PEDOT:PSS can induce inflammatory responses in living cells.^[^ ^129^ ^]^ An efficient way to enhance bio‐functionality is to introduce functional moieties during PEDOT synthesis. In this way, various EDOT‐derivatives, including hydromethyl‐EDOT,^[^ ^139^ ^]^ chloromethyl‐EDOT,^[^ ^140^ ^]^ and carboxy‐EDOT^[^ ^141^ ^]^ were developed. In addition, blending PEDOT with biopolymers including cellulose,^[^ ^142^ ^]^ hyaluronic acid, pectin,^[^ ^143^ ^]^ and guar gum^[^ ^144^ ^]^ can also efficiently enhance the bio‐functionality of PEDOT.

### 3.2 Organic Semiconductors

The development of organic semiconductors capable of transporting both charges and ions has been achieved, in the main, by tuning the molecular structures of conventional organic semiconductors. A high‐performance OMIEC should efficiently transport charge carriers and adequately inject ions. Therefore, the figure of merit considered here is the product (µC*) of charge carrier mobility (µ) and volumetric capacitance (C*).^[^ ^145^ ^]^ In this section, we provide brief introductions to several polymeric *p*‐type and *n*‐type OMIECs. Polymers have advantages over their molecular counterparts, including efficient intra‐ and interchain transport and facile solution processing. For small‐molecule OMIECs, we recommend referring to other relevant literature reviews.^[^ ^63^, ^146^ ^]^

#### 3.2.1 *p*‐Type Semiconductor‐Based OMIECs

**Scheme 2:**
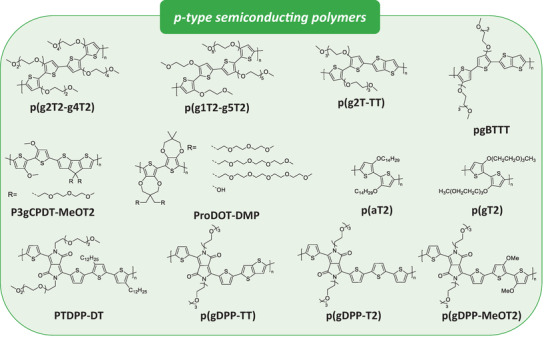
Chemical structures of *p*‐type semiconductor‐based OMIECs.

Most solution‐processable polymers in *p*‐type OMIECs are based on glycolated polythiophene derivatives, as illustrated in **Scheme** **2**. Glycolated polymers exhibit efficient ionic transport due to their hydrophilic nature, while the π‐conjugation maintains electronic charge transport. Since the first demonstration of glycolated polymeric organic semiconductors (OSCs) in 2016,^[^ ^147^ ^]^ many researchers have investigated the effects of glycol side‐chains on different backbones.^[^ ^146^, ^148^ ^]^

A systematic analysis was conducted to investigate the redistribution of glycol chains while maintaining a consistent total number of glycol chains.^[^ ^149^ ^]^ Similar optoelectronic properties were observed with the redistribution, but the µC* values of p(g2T2‐g4T2) and p(g1T2‐g5T2) improved to 496 and 522 F V^−1^ cm^−1^ s^−1^, respectively, with excellent retention in the number of electrochemical switching cycles. Furthermore, the influence of regiochemistry was studied by relocating the glycol side‐chain from 3,3′ (p(g2T‐TT)) to 4,4′ (pgBTTT). pgBTTT exhibited higher backbone planarity due to stronger S─O interaction and consequently resulted in a higher µC* value of 502 F V^−1^ cm^−1^ s^−1^.^[^ ^150^ ^]^ Glycolated cyclopentadithiophene polymers were utilized to obtain a porous microstructure.^[^ ^151^ ^]^ A porous structure has advantages regarding ion migration compared to denser films, and a porous morphology was achieved via a solution processing technique using a binary solvent system. P3gCPDT‐MeOT2 in particular showed a high µC* value of 476 F V^−1^ cm^−1^ s^−1^ along with good mechanical stability in flexible devices. More recently, a new series of propylenedioxythiophene‐based copolymers has been reported, elucidating the roles of side‐chain length and functionality. Investigations with different in situ techniques revealed that a short polar hydroxy functional group (OH‐DMP) is able to achieve facile doping and high C*. Meanwhile, an increasing number of glycol units in side‐chains led to increased solubility, doping kinetics, stability, and µC*. The highest performance of long polar side‐chains is attributed to enhanced hole mobility, rather than a dramatic change in C*. Furthermore, the role of glycolated side‐chains was thoroughly investigated using a molecular dynamics force field, comparing glycolated p(gT2) with alkoxylated p(aT2).^[^ ^152^ ^]^ The authors revealed that while p(aT2) forms a tilted stack with straight interdigitating side‐chains, p(gT2) forms a deflected stack with s‐bend side‐chains. This distinctive stacking arrangement results in different water penetration pathways, occurring through a π‐stack for the former and through a lamellar stack for the latter. Additionally, the meta‐dynamics simulations demonstrated that the maximum length for cation trapping to not occur in the crystalline phase of glycolated bithiophene is triethylene glycol, whereas in tetraethylene glycol, cation trapping is more likely.

Another class of *p*‐type glycolated polymers is based on diketopyrrolopyrrole (DPP) derivatives. DPP, a well‐known electro‐deficient building block, was utilized in electrochemical transistors with two different aqueous electrolytes.^[^ ^153^ ^]^ The BF_4_^−^ based aqueous electrolyte showed high transconductance and a µC* value of 599 F V^−1^ cm^−1^ s^−1^. The authors claimed that the BF_4_^−^ anions have higher doping efficiency, compared to Cl^−^ anions, mostly due to their larger crystallographic radii. A separate series of glycolated DPP derivatives have been documented, namely p(gDPP‐TT), p(gDPP‐T2), and p(gDPP‐MeOT2).^[^ ^154^ ^]^ The authors have elucidated how the distinct microstructures of these three polymers influence µC* performance. Given that C* values are largely comparable, it is essential to align the relative electron density of donor and acceptor fragments to enhance hole mobility. In other words, reducing the energy level disparity between these two fragments spreads hole polaron across larger areas, thereby increasing hole mobility. Consequently, p(gDPP‐TT) and p(gDPP‐T2), which exhibit similar orbital distributions between the donor and acceptor fragments to HOMO, demonstrate higher hole mobility in comparison to p(gDTT‐MeOT2), which has narrower HOMO distributions.

#### 3.2.2 *n*‐Type Semiconductor‐Based OMIECs

**Scheme 3:**
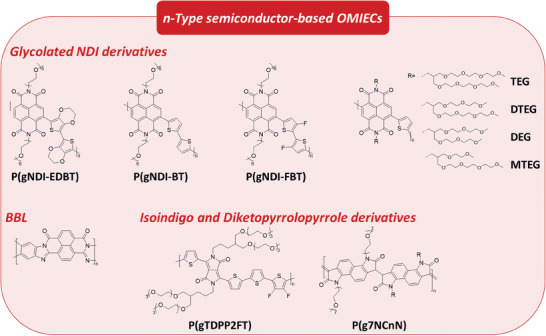
Chemical structures of *n*‐type semiconductor‐based OMIECs.

Despite their importance in integrated circuits, the development of *n*‐type OMIECs lags behind their *p*‐type counterparts due to their ambient instability and low charge carrier mobility. Current research in *n*‐type materials can be classified into three categories: naphthalenediimide (NDI)‐based polymers, poly(benzimidazobenzophenanthroline) (BBL), and isoindigoand DPP‐based polymers (**Scheme** **3**).

Following the first demonstration of an *n*‐type OECT using glycolated NDI derivatives, researchers have focused on developing high‐performance NDI derivatives.^[^ ^86^ ^]^ Three series of glycolated NDI‐based copolymers, namely gNDI‐EDBT, gNDI‐BT, and gNDI‐FBT, were reported with varying donor moieties.^[^ ^155^ ^]^ Among them, gNDI‐FBT, which has a fluorine unit that weakens the electron‐donating ability, exhibited superior electrochemical stability and electron mobility. The enhanced redox stability is expected to arise from more delocalized charges at the bipolaronic state along the backbones, while the high electron mobility is due to preferential molecular packing. Moreover, four glycolated NDI‐based copolymers with varying chain dissymmetry and length were compared.^[^ ^156^ ^]^ P(NDIMTEG‐T), which has the shortest asymmetric side‐chain, showed the highest doping efficiency and electrochemical stability. The authors also demonstrated the use of environmentally friendly ethanol and water as co‐solvents, where they solubilized polar side chains and intensified π‐stacking of the NDI core, thus forming an edge‐on packing. The resultant OECT showed a high µC* value among *n*‐type materials of 0.56 F V^−1^ cm^−1^ s^−1^.

The second class of *n*‐type OMIECs is based on BBL polymers that lack glycolated side chains. A common belief in the field of OMIECs is that hydrophilic side chains are necessary for high performance. However, the BBL polymer with a rigid structure also exhibits mixed conduction.^[^ ^157^ ^]^ Due to its tightly packed edge‐on orientation, BBL showed significantly higher mobility than NDI‐based polymers with glycolated side chains. Surprisingly, BBL also exhibited a threefold higher C* value and negligible swelling in electrolytes. The rigid BBL polymer self‐assembles into crystallites with strong connectivity that resist water uptake while generating ion‐permeable nano‐voids.

The last class of *n*‐type OMIECs was proposed recently and is based on isoindigo and DPP polymers. An isoindigo‐based polymer, p(g7NC10N), showed high *n*‐type performance with a µC* value of 1.83 F V^−1^ cm^−1^ s^−1^.^[^ ^158^ ^]^ Nuanced synthetic considerations are needed to maximize glycol chain length and achieve high‐performance OECT and organic thermoelectric (OTE) devices. Furthermore, a well‐known *p*‐type material, thiophene‐flanked DPP, was transformed to *n*‐type by introducing more charges with a fluorine atom to the donor moiety.^[^ ^159^ ^]^ Through computational analysis, the authors found that the conversion results were not only due to a lower lowest unoccupied molecular orbital (LUMO) level but also from a uniform negative charge distribution, enhanced backbone planarity, and more stable negative polarons after n‐doping. These properties allowed p(gTDPP2FT) to exhibit a record‐high µC* value of 54.8 F V^−1^ cm^−1^ s^−1^.

## 4 Applications

OMIECs are materials that exhibit the unique ability to conduct both ions and electrons. As a result, they are able to be applied to various applications including electrochemical devices, circuits and logic gates, sensors, actuators, OECTs, batteries, fuel cells, electro/photochromic devices, light‐emitting devices, photovoltaics, and so on. In this chapter, we provide an overview of OMIEC‐based applications. In particular, we focus on recent advances in chemical and biosensors; pressure sensors; neural interfacing; neuromorphic devices; and electrochromic devices.

### 4.1 Chemical Sensor and Biosensor

Due to their biocompatibility and long‐term stability in aqueous environments, OECTs have been widely utilized for biosensing applications. In addition, OECT‐based biosensors can amplify the signals from the detection of chemical and biological substances, such as antigens,^[^ ^31^, ^160^, ^161^, ^162^, ^163^ ^]^ neurotransmitters,^[^ ^164^, ^165^, ^166^ ^]^ biomarkers,^[^ ^167^, ^168^, ^169^ ^]^ metabolites,^[^ ^170^, ^171^ ^]^ and even ions,^[^ ^172^, ^173^, ^174^, ^175^ ^]^ through high transconductance.

OECT‐based biosensors are constructed by immobilizing a selective recognition component to the gate electrode or along the channel surface. Specific biochemical interactions induce a change in potential drop or capacitance at the interface between the gate or channel and the electrolyte, which in turn finely tunes the current traversing the channel.^[^ ^176^ ^]^ Biomolecules are categorized into two groups: those that are electroactive and those that are not. Electroactive substances, such as dopamine and serotonin, are capable of being electrochemically oxidized or reduced directly upon the electrode.^[^ ^94^ ^]^ Conversely, electro‐inactive molecules like glucose, lactate, proteins, and nucleic acids necessitate utilizing bio‐recognition methods. Moreover, there are OECT sensors that detect changes in the doping condition of the conductive polymer, influenced by variables like ion concentration and pH levels.^[^ ^177^ ^]^

**Figure 4:**
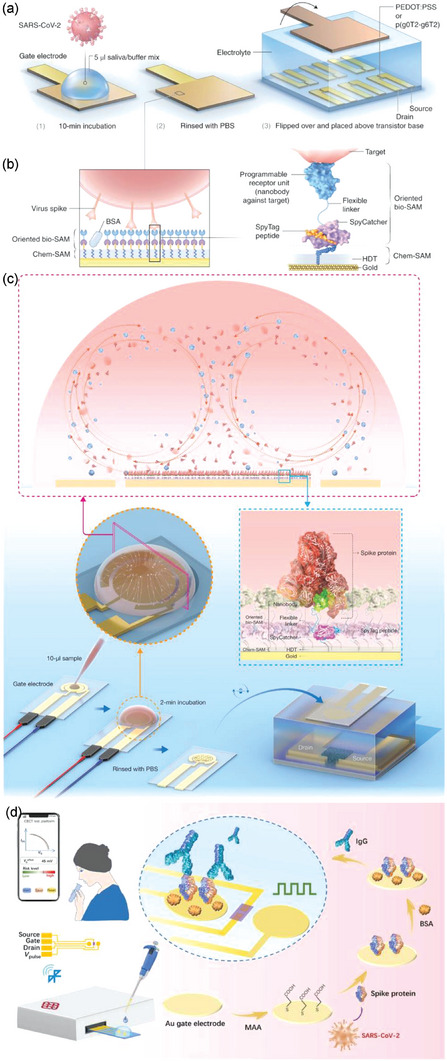
a) Operation process of the nanobody‐functionalized OECT sensor. b) Schematic and molecular architectures of the gate functionalization layer. Reproduced with permission,^[^ ^31^ ^]^ Copyright 2021, Springer Nature. c) Operational and structural schematics of the ACET‐enhanced nanobody‐OECT biosensor. Reproduced with permission,^[^ ^160^ ^]^ Copyright 2022, Wiley‐VCH. d) Schematics of a portable antigen sensing system and the gate functionalization processes of the IgG sensor. Reproduced with permission,^[^ ^163^ ^]^ Copyright 2021, American Association for the Advancement of Science.

Respiratory viruses such as MERS and SARS‐CoV‐2 have dramatically increased the necessity for rapid detection and quantification of antigens.^[^ ^178^ ^]^ To meet that need, Guo et al. reported electrochemical antigen sensing technology, which showed rapid and sensitive quantification of MERS and COVID‐19 antigens through nanobody‐functionalized OECTs with a modular architecture (**Figure** **4**a,b).^[^ ^31^ ^]^ The sensor consisted of PEDOT:PSS or p(g0T2‐g6T2)‐conjugated polymers as a channel, electrolytes, and a nano‐body SpyCatcher‐functionalized gate electrode. The OECT‐based biosensor detected specific antigens from unprocessed complex bodily fluids at single‐molecule‐to‐nanomolar levels after only 10 min of incubation. Interestingly, the sensor can be modified to target any other antibodies if the corresponding nanobody is available. Preliminary tests on patients with COVID‐19 and healthy people demonstrated that the accuracy and sensitivity are comparable to PCR tests. The research group further developed the detection speed and specificity of the platform via adapting alternating current electrothermal flow (ACET) in the OECT sensor (Figure 4c).^[^ ^160^ ^]^ The ACET induced convective vortices in the sample and the mixing reduced the time for immunocomplex formation by fivefold (from 10 to 2 min), supporting its use in clinical practice. In addition, the convective vortices enhanced the specificity by washing nonspecific species away from the sensor surface (from ×10^−18^ to ×10^−9^ m).

As point‐of‐care testing (POCT) is growing in importance for prevention and early screening of disease, many portable devices that can be remotely controlled have been developed.^[^ ^160^ ^]^ Lie et al. reported an OECT‐based ultrafast, low‐cost, and portable SARS‐CoV‐2 immunoglobulin G (IgG) biosensor, which can be remotely controlled by a mobile phone (Figure 4d).^[^ ^163^ ^]^ To reduce detection time, voltage pulses were applied on the gate electrodes of the OECTs, which are modified with SARS‐CoV‐2 spike proteins that can selectively capture the IgG through the specific antibody–antigen reaction, to accelerate immunocomplex formation. The sensor allowed specific detection of SARS‐CoV‐2 IgG in several minutes with a low concentration range (from 10 fM to 100 nM) in saliva and serum samples, and the limit of detection was ≈1 fM in aqueous solutions.

**Figure 5:**
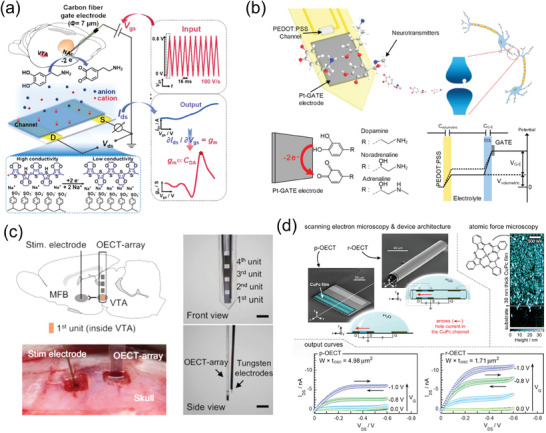
a) Schematic illustration of the FSP‐OECT for in vivo dopamine sensing. The potential waveform is transferred to transconductance as the output signal through the PEDOT:PSS channel. Reproduced with permission,^[^ ^164^ ^]^ Copyright 2022, Wiley‐VCH. b) Operational and structural schematics of an OECT array for CA‐NT detection. c) Schematic illustration and surgical photogram of the in vivo experiments. Reproduced with permission,^[^ ^165^ ^]^ Copyright 2020, eLife Science Publications Ltd. d) Microscopic characterization of self‐curved OECTs and their output characteristics. Reproduced with permission,^[^ ^166^ ^]^ Copyright 2021, Wiley‐VCH.

Neurotransmitters are the words of neuronal language and detecting them is extremely important for understanding how perception, motor control, and cognitive behavior occur.^[^ ^179^, ^180^ ^]^ Detecting and recording various neurotransmitters is valuable for key breakthroughs in medicine, pharmaceuticals, and neurology. Li et al. reported a fast‐scanning potential (FSP)‐gated OECT, that detects dopamine with high sensitivity in a living rat brain (**Figure** **5**a).^[^ ^164^ ^]^ The configuration of the system includes fast‐scan cyclic voltammetry and an OECT, which confers high selectivity and sensitivity to the system, respectively. The origin of the sensitivity is the amplification effect of the OECT, which brings the limit of detection down to the nM‐level for dopamine sensing with desirable reproducibility and stability.

In addition to the biocompatibility and flexibility of OECTs, the low operation voltage of OECTs is advantageous for the detection of neurotransmitters. Compared to conventional cyclic voltammetry, the lower working voltage of OECTs can protect tissue electrolysis during in vivo detection.^[^ ^94^ ^]^ Xie et al. demonstrated a catecholamine neurotransmitter (CA‐NT) (e.g., dopamine, noradrenaline, and adrenaline) monitoring technique in the brains of living animals by using a PEDOT:PSS‐based OECT (Figure 5b).^[^ ^165^ ^]^ The OECT was applied to detect dopamine release in brain regions and showed a desirable limit of detection for dopamine (30 nM) in artificial cerebrospinal fluid containing 1.28 mm of ascorbate (Figure 5c). By modifying the surface of the Pt‐gate electrode with a Nafion‐based multilayer [Nafion/polyaniline (PANI)/enzyme/graphene], the selectivity of the sensor could be enhanced. Furthermore, the target CA‐NT could be changed by choosing the coating enzyme.

Despite the low operation voltage, OECT‐based neurotransmitter sensors are considered not feasible because of the low operation speed due to the time‐consuming doping process and low carrier mobility of OMIECs. To address this issue, Ferro et al. developed the strain‐engineering‐based OECT by using the exclusive shape features of self‐curled nanomembranes (Figure 5d).^[^ ^166^ ^]^ OECTs based on self‐curled nanomembranes (r‐OECTs) feature small‐molecule nanometer‐thick OSCs showed high levels of performance in comparison with their planar staggered counterparts (p‐OECTs), reaching lower operating voltage, improved ion doping, and a signal amplification with high intrinsic gain. The r‐OECT was applied to detect dopamine and showed a limit of detection of (3.04 ± 0.49) × 10^−6^ m, which is comparable with clinical levels.

### 4.2 Pressure Sensor

Many studies on OMIEC‐based physical sensors have been reported to date. OMIECs are great candidates for the active layer or electrodes of a physical sensor due to their high ionic and electronic conductivities. Their capacity to store and transport effectively both ionic and electronic charge makes them promising for various applications. Additionally, they usually exhibit mechanical flexibility, biocompatibility, ease of processing, and stability against chemical and mechanical stimuli. These attractive properties make OMIECs highly efficient in sensing physical properties such as pressure and strain. In terms of a combination of biology and electronics, crucial for bioelectronics, the integration of these sensing properties for various stimuli can significantly advance wearable platforms, healthcare devices, and bioimplantable systems.

**Figure 6:**
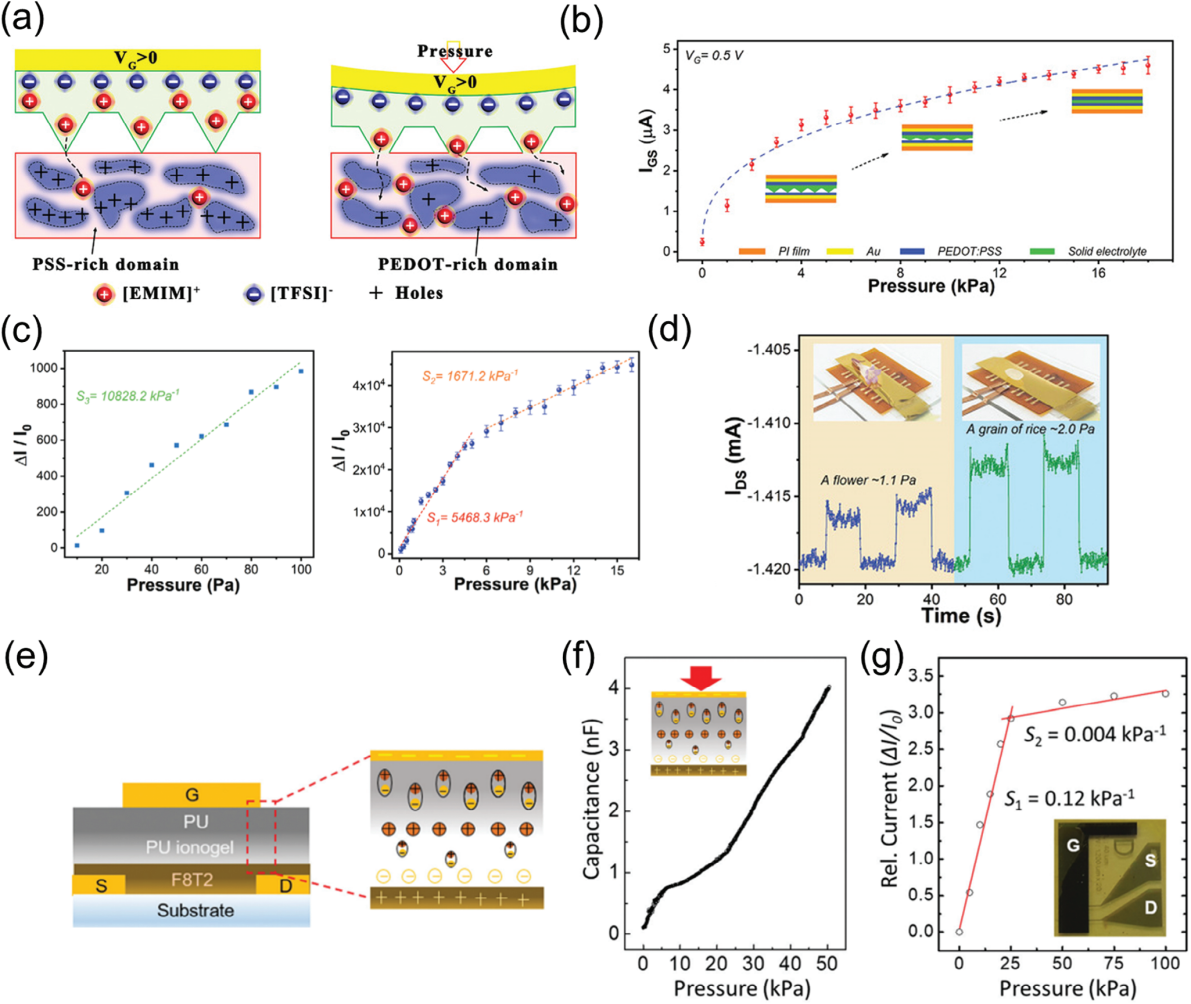
a) Schematic illustration of the contact modulated ionic doping mechanism for pressure sensor. b) The *I*_GS_ under different pressure stimuli at *V*_G_ = 0.5 V. c) Sensitivity to the pressure of the P3HT device at a different pressure region. d) The limit of detection with a flower and a grain of rice. The limit of detection of the OECT‐based pressure sensor was examined at *V*_D_ = −0.5 V and *V*_G_ = 0.6 V. Reproduced with permission.^[^ ^181^ ^]^ Copyright 2020, Wiley‐VCH. e) Schematic diagram of the top‐gate bottom‐contact organic thin‐film transistor (OTFT). Architecture (left) and polarization mechanism of PU ionogel/PU bilayer gate dielectrics (right). f) Pressure response of PIB‐based films using the metal–insulator–metal structure under static pressure conditions at a bias of 0.1 V g) Pressure response of the F8T2/PIB OTFT pressure sensors at *V*_D_ = 2 V and *V*_G_ = 6 V. The inset shows the digital image of the top‐gate bottom‐contact (TGBC) OTFT pressure sensor. Reproduced with permission.^[^ ^183^ ^]^ Copyright 2020, Royal Society of Chemistry.

Pressure sensors composed of OMIECs as an active layer can utilize the mixed ionic and electronic conduction, especially with electrolytes by doping mechanism. For example, Chen et al. reported that PEDOT:PSS with the electrolyte which is the mixture of poly(vinylidene fluoride‐co‐hexafluoropropylene) (PVDF‐co‐HFP) and 1‐ethyl‐3‐methylimidazolium bis(trifluoromethylsulfonyl)amide ([EMIM][TFSI]) showed good performance in a tactile sensor using contact modulated ionic doping mechanism.^[^ ^181^ ^]^ When high pressure was applied to the device with a positive gate voltage, an increase in both the contact area and compressed thickness of the pyramidal structure induced much more capacity at the interface between the electrolyte and the active layer, promoting the migration of a larger quantity of EMIM^+^ cations (**Figure** **6**a). This induced an electrochemical switch in the electron‐conducting PEDOT‐rich domain to a modulated neutral semiconducting state enhancing gate‐source current (Figure 6b). The researchers also employed another OMIEC, P3HT as the channel material, which could be doped by TFSI^−^ anions in the region of side alkyl chain. The resulting all‐solid‐state OECT‐based pressure sensors exhibited a remarkable sensitivity of up to 10 828.2 kPa^−1^ (Figure 6c), excellent stability over a period exceeding 2 months, an outstanding limit of detection of 1.1 Pa which was demonstrated with a flower and rice (Figure 6d) and a low operating voltage ≈1 V with ultra‐low power consumption. Wang et al. similarly demonstrated an OECT‐based iontronic pressure sensor utilizing a microstructured solid hydrogel as the gate dielectric to enhance sensitivity to external pressure.^[^ ^182^ ^]^ Due to the high capacitive nature of the electrical double layer in OECT‐based pressure sensors, they can operate at low voltages with minimal power consumption. In both cases, the device structure was designed to change in response to the applied pressure. As the applied pressure increases, the contact area of the gel at the interface between either the gate electrode and the electrolyte or the electrolyte and the active layer expands. Consequently, the density of mobile ions at the electrolyte/active layer interface increases, thereby enhancing the magnitude of the ionic‐electronic coupling and raising the probability of ion migration through the widened ion migration pathways. This facilitates easier penetration, enabling more ions to be doped and resulting in an increase in the direct ionic–electronic coupling.

Tabi et al. reported the introduction of polyurethane (PU) ionogel/PU bilayer (PIB) over mixed ionic‐electronic conduction in poly(9,9‐dioctylfluorenyl‐2,7‐diyl)‐alt‐co‐(bithiophene) (F8T2) for low‐voltage operated organic transistors and pressure sensors (Figure 6e).^[^ ^183^ ^]^ The application of pressure induces changes in ion concentration within the electric double layers causing a capacitance increase (Figure 6f), driven by the stepwise mechanism involving the viscoelastic deformation of polyurethane chains and the poroelasticity of ionic liquids under mechanical stimuli. As a consequence, both the on‐current and off‐current exhibit an increase under pressure loads. The researchers attribute this increase in current to the penetration and adsorption of anions onto the semiconductor layer. This pressure sensor exhibited a sensitivity of up to 0.12 kPa^−1^ (Figure 6g).

### 4.3 Neural Interfacing

Neural interfaces typically consist of a tissue interface, an electronic sensing interface, and a neural signal processing unit, allowing a direct bridge between nervous systems and man‐made systems.^[^ ^184^ ^]^ Conventionally, the use of metal electrodes or inorganic transistors for neural interfacing has significantly advanced our understanding of actual neural activities.^[^ ^185^ ^]^ The neuronal action potential leads to a flux of ionic currents across the cell membrane, altering the membrane potential.^[^ ^186^ ^]^ This change in potential subsequently generates an electrochemical signal at the metal/electrolyte interface or alters the potential drop across a conducting material.^[^ ^187^ ^]^ However, these metal electrodes present certain challenges, such as biocompatibility and electron transfer at neural interfaces. Because metals primarily conduct electron signals, whereas neural systems transmit ionic signals, the signal‐to‐noise ratio (SNR) in recordings and stimulations performed by bioelectronics largely depends on the interfacial electron‐ion coupling at metal‐neural interfaces.^[^ ^188^ ^]^ Furthermore, the implantation of metal electrodes into neural tissue often triggers an inflammatory response due to mechanical mismatch, which can ultimately lead to electrode degradation in long‐term experiments.^[^ ^189^, ^190^, ^191^ ^]^ To achieve improved neuron‐electrode interfaces, recent developments in bioelectronics have focused on OMIECs. These OMIEC‐based electrodes and transistors have demonstrated enhanced capabilities in neural recording and stimulation.^[^ ^192^ ^]^ Their advantages include mixed conduction capabilities, biocompatibility, and lower mechanical strength, which are critical for effective and sustainable integration with neural tissues.

**Figure 7:**
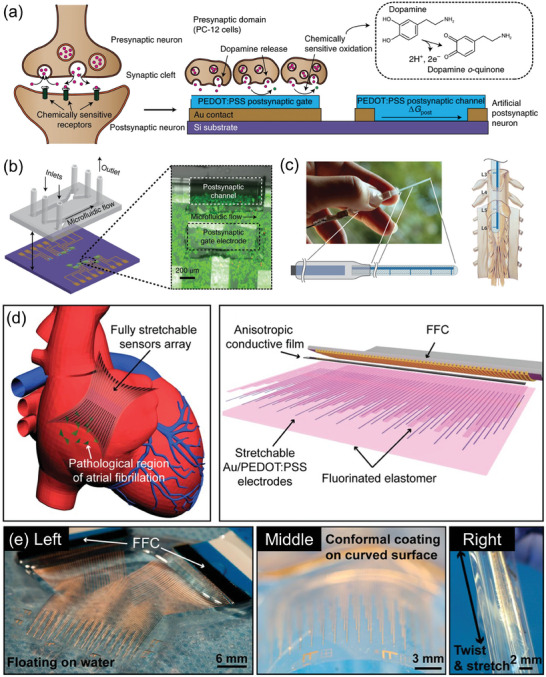
a) Operational schematics of a biological synapse and a neurotransmitter‐mediated neuromorphic device b) Schematics and operation of a neuromorphic device with a microfluidic channel. Synaptic plasticity was measured using source–drain conductance. Reproduced with permission,^[^ ^194^ ^]^ Copyright 2020, Springer Nature. c) Photograph of the implantable organic electronic ion pump (OEIP) and schematic of an implanted OEIP in the human spinal cord. Reproduced with permission,^[^ ^195^ ^]^ Copyright 2015, American Association for the Advancement of Science. d) Schematics of the elastrode array for atrial fibrillation mapping. e) Photographs of the elastrode array with ultralight weight (left), high flexibility (middle), and stretchability (right). Reproduced with permission,^[^ ^196^ ^]^ Copyright 2020, National Academy of Sciences.

Neuromorphic computing and platforms are representative candidates and applications for neural interfacing. In bioelectronics, to reach the full potential of neuromorphic systems, achieving synaptic conditioning based on biochemical signaling activity is necessary.^[^ ^193^ ^]^ Keene et al. demonstrated the convergence of an organic neuromorphic device and dopaminergic cells (PC‐12 cells) to demonstrate a biohybrid synapse with neurotransmitter‐mediated synaptic plasticity (**Figure** **7**a,b).^[^ ^194^ ^]^ The presynaptic domain of PC‐12 cells made contact with the PEDOT:PSS‐based organic neuromorphic device, which acted as the postsynaptic domain. In this biohybrid system, dopamine exocytosed by PC‐12 cells at the presynaptic end is locally oxidized at the postsynaptic gate electrode of the neuromorphic device. By mimicking the dopamine‐recycling machinery of the synaptic cleft, they demonstrate both long‐term plasticity and recovery of the synaptic weight.

Another application of neural interfacing is in vivo therapy or the recording of signals using implanted electronics. Direct therapeutic action with implantation can be conducted at specific sites in the body, and many side effects can be minimized compared to drug therapy.^[^ ^184^, ^189^ ^]^ Jonsson et al. utilized PEDOT:PSS to design an implantable device for local electrically controlled delivery of therapeutics (Figure 7c).^[^ ^195^ ^]^ The PEDOT:PSS induced ionic and molecular fluxes by transducing electronic pulses into biological signals. Implantable devices have been showcased for precise administration of substances to neural tissue, as well as for biosensing, recording, and stimulating neural activity. The devices were surgically inserted into the spinal cord of rats. Two days post‐implantation, the controlled release of the inhibitory neurotransmitter g‐aminobutyric acid (GABA) was initiated. The precise and targeted delivery led to a notable reduction in pain response, achieved with a minimal dose and without detectable adverse reactions.

Electrophysiological cardiac mapping is critical for understanding and early screening of heart disease, but conventional electrophysiological tools have limitations of spatial resolution and electromechanical uncoupling on the beating heart. To address these issues, Liu et al. developed an Au/PEDOT:PSS‐based fully stretchable and encapsulated elastic electrode array (Figure 7d,e).^[^ ^196^ ^]^ The device showed robust and intimate tissue coupling for acute rabbit and porcine models and maintained intrinsic properties during the cardiac cycle. The electrode array showed high‐performance levels including a twofold higher atrial‐to‐ventricular signal ratio and a >100‐fold higher spatial resolution with comparable efficacy to currently available endocardial‐mapping techniques.

### 4.4 Neuromorphic and Memory Devices

In traditional von Neumann computing architecture, where the main memory unit and central processing unit (CPU) are distinct entities, a substantial amount of energy is consumed during complex data processing. Software‐based artificial neural networks, represented by AlphaGo, borrowed software algorithms based on deep learning technology and data processing methods using big data. This means that a huge number of hardware devices such as CPUs, graphic processing units, and dynamic random access memories are required, necessitating energy consumption that is too high to be applied to mobile devices or the Internet of Things. Neuromorphic devices have thus attracted significant attention as technologies that can break through this bottleneck.^[^ ^197^ ^]^

Many different kinds of OECT‐based neuromorphic and memory devices using OMIECs as an active layer have been developed. The first demonstration of a neuromorphic device based on a PEDOT:PSS OECT that emulates certain functions in the biological neural system, such as paired‐pulse depression, adaptation, and dynamic filtering, was reported in 2015.^[^ ^198^ ^]^ Generally, an attractive figure‐of‐merit of neuromorphic devices is their potential for lower energy consumption per switching event. This was demonstrated in devices utilizing polymer nanofibers as the active layer which exhibit remarkably low energies reaching as low as 1.23 fJ per synaptic spike.^[^ ^199^ ^]^

**Figure 8:**
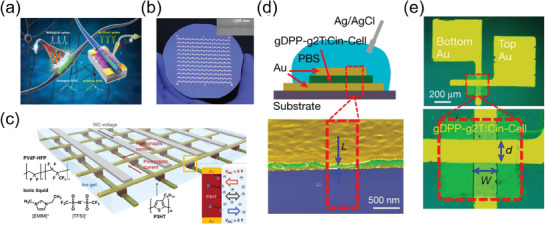
a) Schematic illustrations of a biological axon and an organic nanowire‐based synaptic transistor (ONWST) that mimics a biological axon. b) Array of 144 ONWSTs on a 4‐inch Si wafer. Reproduced with permission,^[^ ^199^ ^]^ Copyright 2016, American Association for the Advancement of Science. c) Schematic illustration of an array of ion‐gel‐gated 3D vertical crossbar synaptic transistors. Reproduced with permission,^[^ ^202^ ^]^ Copyright 2020, Springer Nature. d) Cross‐section illustration of *p*‐type vertical OECTs. e) Optical microscopy image of a *p*‐type vertical OECT. Reproduced with permission,^[^ ^204^ ^]^ Copyright 2023, Springer Nature.

As previously mentioned, during the initial stages of development of this field, numerous researchers focused on reducing energy consumption per synaptic spike, enhancing synaptic characteristics including short‐term and long‐term plasticity, and developing non‐volatile memory devices. However, in recent years, efforts to encompass the applications of integrated circuits, the emulation of more complex functions of biological neural networks, and applications to biomedical engineering have expanded their focus.^[^ ^34^, ^200^, ^201^ ^]^ To enable a denser array, device miniaturization plays a crucial role. Xu et al. achieved this by employing a core–sheath polymer nanofiber structure, where the core consisted of P3HT and the sheath was made of polyethylene oxide (PEO) and successfully fabricated a large‐scale array comprising 144 synaptic transistors on a 4‐inch‐diameter wafer (**Figure** **8**a,b). However, both planar and in‐plane gate OECTs are likely to face certain structural limitations, particularly in terms of low array density and complex line design. To address these limitations, a novel design for a vertical OECT‐based neuromorphic architecture has been demonstrated. A vertical organic synapse array, composed of vertically stacked electrodes, with nanoscale channel length was successfully fabricated by Choi et al. using azide photocrosslinker bis(6‐((4‐azido‐2,3,5,6‐tetrafluorobenzoyl)oxy)hexyl)decanedioate for the crosslinking reaction of the channel material (Figure 8c).^[^ ^202^ ^]^ Despite the remarkable potential of complementary circuits based on OECTs in the field of bioelectronics, their practical implementation has been challenging due to issues such as the operational instability and inferior performance of *n*‐type OECTs.^[^ ^86^, ^104^, ^203^ ^]^ By blending redox‐active semiconducting polymers with a redox‐inactive photopatternable polymer, *p*‐ and *n*‐type vertical OECTs for complementary circuits and inverters were successfully demonstrated recently as shown in Figure 8d,e.^[^ ^204^ ^]^ For the *p*‐type OECT channel material, the authors used gDPP‐g2T, while for the *n*‐type OECT channel material, homo‐gDPP was utilized. In addition, the photocrosslinker Cin‐cell was employed in the fabrication process. These circuits demonstrated footprint current densities exceeding 1 kA cm^−2^ at voltages of less than ±0.7 V. They also exhibited transconductances ranging from 0.2 to 0.4 S, short transient times less than 1 ms, and remarkably stable switching (>50 000 cycles). This innovative design was able to overcome previous limitations and offer enhanced performance and reliability for complementary logic OECTs. Despite these remarkable developments, a challenge still remains when the electrolyte layer is not patterned, resulting in all of the neuromorphic devices being interconnected through the electrolyte. Consequently, the occurrence of unwanted leakage currents and crosstalk between neighboring devices becomes inevitable. To overcome this issue and realize a high array density, the implementation of a patternable solid electrolyte is necessary.^[^ ^205^ ^]^

**Figure 9:**
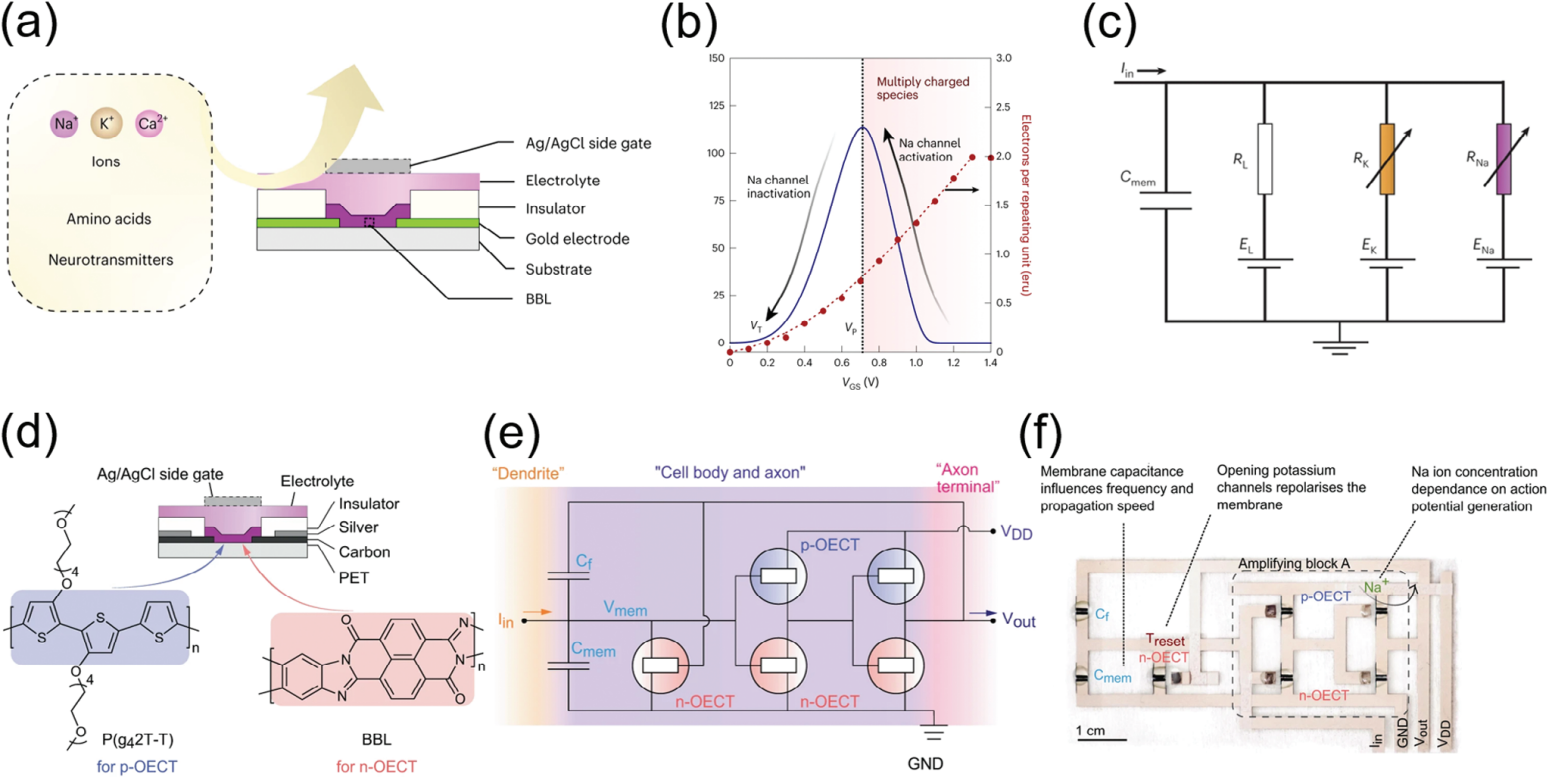
a) Schematic illustration of an OECT device structure. b) The stable inverted‐V response characteristic (anti‐ambipolar behavior) in BBL. c) The Hodgkin‐Huxley circuit of the OECNs. Reproduced with permission,^[^ ^206^ ^]^ Copyright 2023, Springer Nature. d) Schematic illustration of the printed OECTs and chemical structures of *p*‐type P(g42T‐T) and *n*‐type BBL. e) Schematic illustration of an OECN‐based Axon–Hillock circuit. f) Photograph of fully printed OECNs. Reproduced with permission,^[^ ^208^ ^]^ Copyright 2023, Springer Nature.

A major step forward in demonstrating the potential of OMIECs as neuromorphic materials was the use of an anti‐ambipolar OECT based on the conjugated ladder‐type polymer OMIECs, BBL, to emulate various biological neural functions. The stable inverted‐V response characteristic of the anti‐ambipolar transistor opened up a new method for novel circuit designs, as it can replicate the activation‐inactivation properties of biological synapses. Utilizing BBL, the authors successfully demonstrated a neuromorphic device called the conductance‐based organic electrochemical neuron (c‐OECN) (**Figure** **9**a–c).^[^ ^206^, ^207^ ^]^ The c‐OECN emulated the activation/inactivation behavior of sodium channels and delayed activation of potassium channels observed in biological neurons. Remarkably, the c‐OECN exhibited many features similar to biological neurons, operating at frequencies approaching 100 Hz, which is comparable to ion‐based neurons found in biology. The devices and circuit fabricated here enabled the effective demonstration of various functions of biological neurons including controlling spiking activity using common biological ions (Na^+^, K^+^, and Ca^2+^), spike inhibition mediated by neurotransmitters, and spike‐dependent threshold voltage shifts. These characteristics highlighted the significant progress achieved by the c‐OECN.^[^ ^206^, ^207^ ^]^ Furthermore, the authors reported the first organic electrochemical neurons (OECNs) capable of ion‐modulated spiking. These OECNs were developed using all‐printed complementary OECTs, consisting of a *p*‐type OECT made of glycolated polythiophene (P(g42T‐T)) as the hole‐transporting material and an *n*‐type OECT composed of BBL as the electron‐transporting material (Figure 9d–f). The Axon–Hillock circuit was selected by the authors as the basis for fabricating a leaky integrate and fire (LIF) type spiking OECN. Notably, the integration of OECNs with the Venus flytrap plant (*Dionaea muscipula*) was successfully demonstrated, resulting in the closure of lobes in response to external stimuli. This bio‐integration clearly demonstrated the compatibility of OECNs with biological systems.^[^ ^208^ ^]^

Artificial synapses have the capability to mimic the functions of biological synapses and can be utilized as substitutes for impaired sensorimotor functions in biological systems. Additionally, similar to the sensory‐reception mechanism found in biological systems, neuromorphic devices have the ability to receive sensory information through bioinspired sensory receptors. To duplicate the sensory and motor functions of their biological counterparts, many studies on combining sensors and actuators with neuromorphic devices have been conducted. These efforts were aimed at achieving a comprehensive integration of artificial systems with biological systems, facilitating the development of advanced functionalities in bioelectronics.

**Figure 10:**
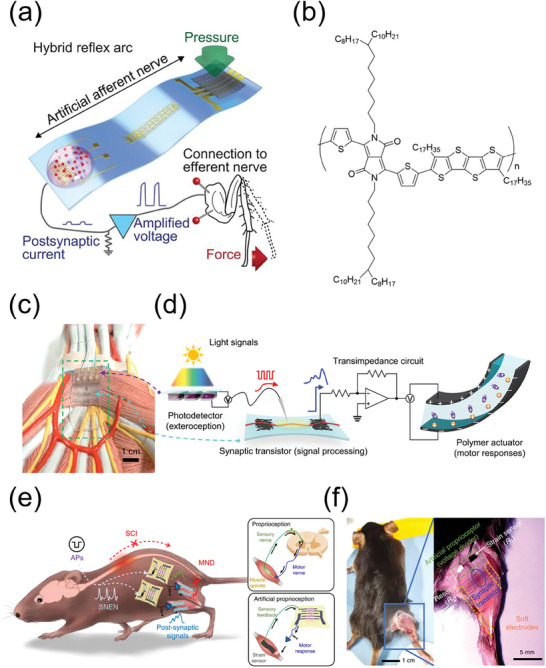
a) Hybrid reflex arc composed of a biological efferent nerve and an artificial afferent nerve. b) Chemical structure of the FT4‐DPP‐based conjugated polymer. Reproduced with permission,^[^ ^209^ ^]^ Copyright 2018, American Association for the Advancement of Science. c) Photograph of *s*‐ONWST on an internal human structure model. d) Schematic illustration of an artificial optoelectronic synapse and a neuromuscular electronic system. Reproduced with permission,^[^ ^215^ ^]^ Copyright 2018, American Association for the Advancement of Science. e) Schematic illustration of a stretchable neuromorphic efferent nerve (SNEN) that can replace damaged nerves. f) Photographs of a mouse with a SNEN attached to its leg. Reproduced with permission,^[^ ^216^ ^]^ Copyright 2022, Springer Nature.

Lee et al. conducted various studies, starting with the development of neuromorphic devices that can replace the peripheral nervous system in 2018. An artificial afferent nerve, composed of pressure sensors, an organic ring oscillator, and a synaptic transistor stimulated by pressure were demonstrated (**Figure** **10**a).^[^ ^209^ ^]^ In this study, the authors utilized a fused thiophene (FT4)‐DPP‐based conjugated polymer, poly[(3,7‐bis(heptadecyl)thieno[3,2‐b]thieno[2′,3′,:4,5]thieno[2,3‐d]thiophene‐5,5′‐diyl)(2,5‐bis(8‐octyloctadecyl)−3,6‐di(thiophen‐2‐yl)pyrrolo[3,4‐c]pyrrole‐1,4(2H,5H)‐dione‐5,5′‐diyl)], with a short decay time (2.35 ms) and small hysteresis to develop their artificial afferent nerves. The decay time of synaptic responses varies across different biological organs (Figure 10b).^[^ ^210^, ^211^, ^212^, ^213^ ^]^ Therefore, tuning the decay time of artificial synapses to suit specific applications in bioinspired electronics is necessary. By adjusting the decay time, artificial synapses can mimic the behavior of their biological counterparts more accurately and effectively transfer information in a manner that is tailored to the intended use or functionality of the bioinspired electronic system. This tunability enables the optimization of artificial synapses for specific applications, enhancing their performance and functionality in neuromorphic devices.^[^ ^214^ ^]^ However, when using P3HT synaptic transistors, the presence of significant hysteresis resulted in a relatively long decay time (299 s), which led to a high initial current level after applying pressure. Furthermore, stretchable organic nanowire synaptic transistors (s‐ONWSTs) were developed using electrospun single nanowires based on both the FT4‐DPP‐based conjugated polymer and PEO (Figure 10c,d).^[^ ^215^ ^]^ The highly robust s‐ONWSTs exhibited stable I‐V characteristics and various typical synaptic characteristics such as excitatory postsynaptic current (EPSC), paired‐pulse facilitation (PPF), spike‐voltage‐dependent plasticity (SVDP), spike‐number‐dependent plasticity (SNDP), and spike‐frequency‐dependent plasticity (SFDP) at both 0% and 100% strains. By establishing a connection between an artificial muscle and artificial efferent nerves based on s‐ONWSTs, it became possible to control the movement of the artificial muscles in a biomimetic manner. Moreover, the authors successfully demonstrated the connection between artificial efferent nerves based on s‐ONWSTs and biological muscles, aiming to enhance limb movement in cases of nerve damage caused by spinal cord injuries or lower motor neuron impairments (Figure 10e,f).^[^ ^216^ ^]^ This research showed that the artificial efferent nerves could restore motions in the legs of mice with neurological motor disorders. For more comprehensive information on neuromorphic devices, from neural interfaces to neuroprosthetics, we recommend relevant literature reviews.^[^ ^201^ ^]^

### 4.5 Electrochromic Devices

Due to their ability to readily undergo redox reactions, OMIECs are promising candidates for use in electrochromic devices (ECDs) as their optical changes can result in coloration. The pristine device is formed by unique color OMIECs, which are in the neutral states, determined by their optical bandgaps. In the case of the OMIEC based on *p*‐type conjugated polymer (or *n*‐type), applying a negative bias (or a positive bias) to the counter electrode of the device injects anions (or cations) of the electrolyte into the *p*‐type OMIEC (or the *n*‐type OMIEC), which are then compensated and stabilized by direct electrostatic ionic–electronic coupling. When charges are induced by that process, the neutral state conjugated polymer turns to single‐charged (polaron) and double‐charged (bipolaron) states, and therefore the bandgap changes the states (bleaching process).^[^ ^217^, ^218^, ^219^, ^220^ ^]^ The coloration process is obtained by applying a positive bias. This alteration changes the conductivity of OMIECs by several orders of magnitude and enables the modulation of optical properties.

In the field of ECDs, there are a few important parameters for assessing the material, including contrast ratio, coloration efficiency, switching time, and cycle stability.^[^ ^221^ ^]^ The contrast ratio is defined as the ratio of transmittance in two redox states, and it depends on the nature of the materials, film thickness, and morphology. In OMIECs, the optical absorbance changes are determined by the magnitude of the ionic–electronic coupling. Coloration efficiency quantifies how effectively an ECD can change color when the potential is applied, determined by the slope of the absorbance change versus charge density. OMIECs usually exhibit good coloration efficiency, particularly poly(dioxithiophenes), which are effective, showing values of 300−500 C^−1^ cm^2^.^[^ ^222^ ^]^ Regarding switching time, which is related to ionic transport, most bioelectronic ECD devices do not require extremely fast switching, so a few seconds are typically sufficient. Cycle stability is a key practical concern when applying OMIECs to ECDs, which can be determined by the number of cycles possible without a loss of optical contrast and charge density. An ideal ECD should demonstrate minimal degradation when exposed for prolonged periods to oxygen and room light.

**Figure 11:**
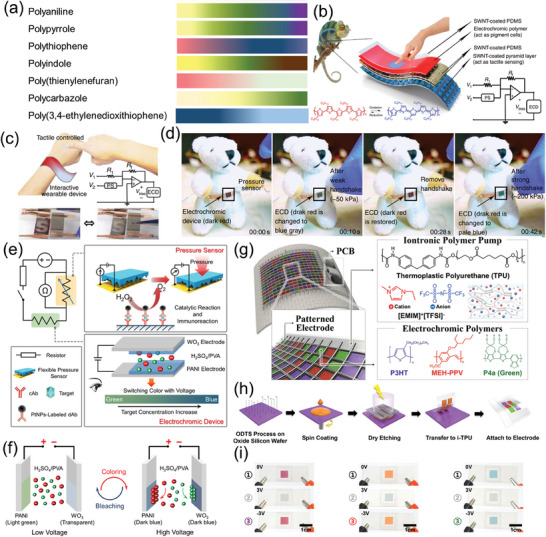
a) Summary of widely used OMIECs and their color window from reduced form to oxidized form. Reproduced with permission,^[^ ^228^ ^]^ Copyright 2021, Elsevier. b) Device structure of a chameleon‐inspired e‐skin, showing the redox states of P3HT and describing the circuit. c) Demonstration of a tactile‐controlled interactive wearable device, showing the color change from dark red to pale blue during the transition from the neutral to oxidized state. d) Interactive color change by sensing the pressure of a strong and weak handshake. Reproduced with permission,^[^ ^230^ ^]^ Copyright 2015, Springer Nature. e) Schematic illustrations of the pressure‐based immunoassay platform consisting of a platinum nanoparticle‐triggered immunoassay with a flexible pressure sensor and a voltage‐regulated electrochromic device. f) Operation mechanism of the electrochromic device with PANI and WO_3_. Reproduced with permission,^[^ ^231^ ^]^ Copyright 2021, American Chemical Society. g) Schematic illustration of the DMECS and the chemical structures of its associated materials. h) Schematic illustration of the fabrication procedure of the DMECS. i) Photographs of the electrochromic materials in initial, bleached (+3 V), and recolored (−3 V) states. Reproduced with permission,^[^ ^232^ ^]^ Copyright 2020, Elsevier.

In order to meet the specified parameters, researchers have employed a range of OMIECs in ECDs, including derivatives of polyaniline, polypyrrole, polythiophene, polyindole, poly(thienylenefuran), polycarbazole, and poly(3,4‐ethylenedioxithiophene) and the color change and contrast depending on the bandgap (**Figure** **11**a). For more detailed descriptions of each material, we recommend referring to other review papers.^[^ ^221^, ^223^, ^224^, ^225^, ^226^ ^]^

Recently, ECDs have found extensive utility within the bioelectronic community, serving as transducer components in various biosensors.^[^ ^227^, ^228^, ^229^ ^]^ The integration of ECDs with biosensors facilitates an intuitive readout through the color change of analytical results, establishing them as a promising platform for point‐of‐care applications. They show a superior trait in terms of low power consumption compared to other displays. This is attributed to their low self‐discharge rate and the fact that they consume energy only during the process of refreshing the displayed content. Among them, ECDs composed of OMIECs offer distinct advantages, including low cost, facile solution processing, and flexibility.

One notable endeavor is the integration of pressure sensors with ECDs. For instance, an e‐skin with electrochromic and tactile sensing properties, inspired by chameleons, has been reported (Figure 11b).^[^ ^230^ ^]^ The stretchable electronic skin was made possible by utilizing P3HT, which undergoes a color change through redox reactions, for electrochromism. Additionally, a tactile sensing layer was created by spray‐coating single‐wall carbon nanotubes with a pyramid layer. Through constructing an integrated circuit consisting of a pressure sensor and a flexible ECD, an interactive e‐skin was fabricated capable of changing color and sensing touch (Figure 11c). The performance of the device was demonstrated by connecting it to a teddy bear where the pressure sensor detected weak and strong handshakes, and the results were expressed through color changes in the ECD (Figure 11d). Furthermore, another study reported a portable pressure‐based immunoassay by integration of pressure sensors and ECD (Figure 11e).^[^ ^231^ ^]^ The carcinoembryonic antigen served as the target, binding with the capture antibody to form an immunocomplex with platinum nanoparticle‐labeled detection antibody. The catalytic capability of platinum nanoparticles converted H_2_O_2_ to O_2_, leading to an increase in pressure due to the excess O_2_. This rise in pressure was then detected by a flexible pressure sensor, resulting in a decrease in resistance. Within the circuit, the voltage signal regulated an ECD based on polyaniline (PANI) and tungsten oxide (WO_3_). At a low voltage, sulfate ions and protons were freely mobile in the gel electrode, whereas at a high voltage, these ions became intercalated in the PANI and WO_3_ electrodes (Figure 11f). Consequently, as the voltage transitions from low to high, the redox state of the two electrodes changes, causing PANI to shift from a light green (the bleached state) to a dark blue(the colored state), while WO_3_ also exhibits a transition from transparent to dark blue.

Another important endeavor inspired by the cephalopod demonstrated an active camouflage application based on a dynamic multicolor electrochromic skin (DMECS) (Figure 11g).^[^ ^232^ ^]^ P3HT, MEH‐PPV, and P4a as electrochromic materials were used to create purple, orange, and green colors, respectively. The flexible DMECS was made using P3HT, MEH‐PPV, and P4a as the electrochromic material to create purple, orange, and green colors, respectively. A freestanding ion gel, composed of [EMIM][TFSI] ionic liquid and thermoplastic polyurethane (TPU) used as the polymer network, was fabricated by spin‐coating. The electrochromic layer was fabricated by spin‐coating onto an octadecyltrichlorosilane (ODTS)‐treated Si wafer and then subjected to dry etching for patterning the electrochromic layers. The patterned electrochromic layer was transferred to the freestanding ion gel. This electrochromic layer‐incorporated ion gel was sandwiched between two flexible electrodes. One was an ITO‐PET as the top electrode and the other was a thermally evaporated patterned Cr/Au‐PET as the bottom electrode.

Additionally, a tactile sensing layer was created by spray‐coating single‐wall carbon nanotubes with a pyramid layer. Through constructing an integrated circuit consisting of a pressure sensor and a flexible ECD, an interactive e‐skin was fabricated capable of changing color and sensing touch (Figure 11c). The performance of the device was demonstrated by connecting it to a teddy bear where the pressure sensor detected weak and strong handshakes, and the results were expressed through color changes in the ECD (Figure 11d). When a voltage of +3 V was applied to the electrochromic layers, the broad absorption maximum, which was observed for each material before applying a positive bias or at 0 V, almost decreased and the electrochromic layers of the device turned into fully bleached states. Upon applying a reverse bias (−3 V), the electrochromic layer of the device almost perfectly returned to its original coloration state. Furthermore, when there was no applied bias (0 V), the colors did not change back to their initial states (i.e., P3HT: purple, MEH‐PPV: orange, P4a: green). The operation mechanism of bleaching/coloration states could be explained as follows: At a positive bias applied to the electrochromic layer, electrons were extracted from the electrochromic polymers and flew moved toward the ITO electrode, while the TFSI^−^ anions were migrated from the ion gel into the electrochromic polymers to keep charge neutrality. On the other hand, applying a negative bias to the electrochromic layer injected electrons, while TFSI^−^ anions were extracted from the electrochromic layers.

## 5 Conclusion and Outlook

Over the years, OMIECs have emerged as potential candidates in a variety of applications due to their unique charge transport abilities which can support both ion and electron transport and even coupled ion–electron transfer. With these properties, OMIECs have been actively applied to organic bioelectronics including (multi)sensors capable of detecting various stimuli, neuromorphic devices, and electrochromic devices. Therefore, a comprehensive focus on the properties and applications of OMIECs benefits the progress in various fields.

In this review, we focused first on the basic physics of ionic and electronic transport, ionic–electronic coupling, and the processes in OECTs, which are important for understanding operation mechanisms for applications. Electronic transport is known to be intermediate between hopping transport in the amorphous phase and band‐like transport in the ordered phase of conjugated polymers. Ionic currents can flow by moving ions within the ion‐conducting materials in the way of ion hopping via segmental motion or ion cluster, solvated/vehicle mechanisms, and the Grotthuss mechanism. Although the mechanism of ionic–electronic coupled transport remains unclear, it plays an important role in the performance and functionality of OMIECs. Therefore, many efforts have been made to clarify the mechanisms of ionic–electronic coupling. Unlike conventional systems, it is driven by interactions between ions and conjugated polymers, and it influences macroscopic physical responses in various applications such as OECT‐based devices. Understanding and optimizing this coupling is essential for advancing the development of OMIECs for different purposes. Many OMIECs are synthesized with PEDOT derivatives as the electrical conductor, including the most well‐known, PEDOT:PSS. Recent research achievements in OMIEC‐based bioelectronic applications include not only independent devices but also integrated or connected systems for high sensitivity and detection capabilities for diverse stimuli. These devices can be applied to advanced bioelectronics such as implantable, wearable, and flexible devices, as well as energy storage devices.

To bring out the full potential of OMIECs and realize their possibilities, it is imperative for future research endeavors to concentrate on comprehending the underlying mechanisms governing ionic and electronic transport within OMIECs. This involves optimizing material characteristics and device structures, exploring innovative synthesis techniques, and establishing dependable fabrication procedures. Collaboration among researchers from diverse fields such as materials science, chemistry, electronics, and biology will be essential to fully unlock the capabilities of OMIECs and propel their integration into advanced technologies of the future.

## Conflict of Interest

The authors declare no conflict of interest.

## References

[1] I. Riess , Solid State Ion 2003, 157, 1.

[2] J. Maier , Nat. Mater. 2005, 4, 805. 16379070 10.1038/nmat1513

[3] Y. Lin , S. Fang , D. Su , K. S. Brinkman , F. Chen , Nat. Commun. 2015, 6, 6824. 25857355 10.1038/ncomms7824 PMC4403342

[4] V. Coropceanu , J. Cornil , D. A. Da Silva Filho , Y. Olivier , R. Silbey , J.‐L. Brédas , Chem. Rev. 2007, 107, 926. 17378615 10.1021/cr050140x

[5] P. Batail , Chem. Rev. 2004, 104, 4887. 10.1021/cr030645s 15535654

[6] H. Jiang , P. Taranekar , J. R. Reynolds , K. S. Schanze , Angew. Chem., Int. Ed. 2009, 48, 4300. 10.1002/anie.200805456 19444838

[7] D. Moia , A. Giovannitti , A. A. Szumska , I. P. Maria , E. Rezasoltani , M. Sachs , M. Schnurr , P. R. F. Barnes , I. Mcculloch , J. Nelson , Energy Environ. Sci. 2019, 12, 1349.

[8] J. Jang , J. Ha , J. Cho , Adv. Mater. 2007, 19, 1772.

[9] K. A. Ludwig , J. D. Uram , J. Yang , D. C. Martin , D. R. Kipke , J. Neural Eng. 2006, 3, 59. 16510943 10.1088/1741-2560/3/1/007

[10] J. Rivnay , S. Inal , B. A. Collins , M. Sessolo , E. Stavrinidou , X. Strakosas , C. Tassone , D. M. Delongchamp , G. G. Malliaras , Nat. Commun. 2016, 7, 11287. 27090156 10.1038/ncomms11287 PMC4838877

[11] J. T. Friedlein , R. R. Mcleod , J. Rivnay , Org. Electron. 2018, 63, 398.

[12] R. Wu , M. Matta , B. D. Paulsen , J. Rivnay , Chem. Rev. 2022, 122, 4493. 35026108 10.1021/acs.chemrev.1c00597

[13] M. Nikkhah , J. Rivnay , Acta Biomater. 2022, 139, 1. 35105464 10.1016/j.actbio.2022.01.018

[14] C. Cendra , A. Giovannitti , A. Savva , V. Venkatraman , I. Mcculloch , A. Salleo , S. Inal , J. Rivnay , Adv. Funct. Mater. 2019, 29, 1807034.

[15] T. J. Quill , G. Lecroy , A. Melianas , D. Rawlings , Q. Thiburce , R. Sheelamanthula , C. Cheng , Y. Tuchman , S. T. Keene , I. Mcculloch , R. A. Segalman , M. L. Chabinyc , A. Salleo , Adv. Funct. Mater. 2021, 31, 2104301.

[16] J. Tropp , D. Meli , J. Rivnay , Matter 2023, 6, 3132.

[17] N. Turetta , M.‐A. Stoeckel , R. F. De Oliveira , F. Devaux , A. Greco , C. Cendra , S. Gullace , M. Gicevicius , B. Chattopadhyay , J. Liu , G. Schweicher , H. Sirringhaus , A. Salleo , M. Bonn , E. H. G. Backus , Y. H. Geerts , P. Samorì , J. Am. Chem. Soc. 2022, 144, 2546. 35129329 10.1021/jacs.1c10119

[18] B. D. Paulsen , K. Tybrandt , E. Stavrinidou , J. Rivnay , Nat. Mater. 2020, 19, 13. 31427743 10.1038/s41563-019-0435-z

[19] A. E. Javier , S. N. Patel , D. T. Hallinan , V. Srinivasan , N. P. Balsara , Angew. Chem., Int. Ed. 2011, 50, 9848. 10.1002/anie.201102953 21901803

[20] S. N. Patel , A. E. Javier , G. M. Stone , S. A. Mullin , N. P. Balsara , ACS Nano 2012, 6, 1589. 22324447 10.1021/nn2045664

[21] A. Malti , J. Edberg , H. Granberg , Z. U. Khan , J. W. Andreasen , X. Liu , D. Zhao , H. Zhang , Y. Yao , J. W. Brill , I. Engquist , M. Fahlman , L. Wågberg , X. Crispin , M. Berggren , Adv. Sci. 2016, 3, 1500305. 10.1002/advs.201500305 PMC5063141 27774392

[22] D. Melling , J. G. Martinez , E. W. H. Jager , Adv. Mater. 2019, 31, 1808210. 10.1002/adma.201808210 30907471

[23] Q. Pei , G. Yu , C. Zhang , Y. Yang , A. J. Heeger , Science 1995, 269, 1086. 17755530 10.1126/science.269.5227.1086

[24] O. Y. Kweon , M. Y. Lee , T. Park , H. Jang , A. Jeong , M.‐K. Um , J. H. Oh , J. Mater. Chem. C 2019, 7, 1525.

[25] J. Isaksson , P. Kjäll , D. Nilsson , N. Robinson , M. Berggren , A. Richter‐Dahlfors , Nat. Mater. 2007, 6, 673. 17643105 10.1038/nmat1963

[26] Y. Van De Burgt , E. Lubberman , E. J. Fuller , S. T. Keene , G. C. Faria , S. Agarwal , M. J. Marinella , A. A. Talin , A. Salleo , Nat. Mater. 2017, 16, 414. 28218920 10.1038/nmat4856

[27] N. L. Nozella , J. V. M. Lima , R. F. De Oliveira , C. F. D. O. Graeff , Mater. Adv. 2023, 4, 4732.

[28] S. Hazra , A. Banerjee , A. K. Nandi , ACS Omega 2022, 7, 32849. 36157781 10.1021/acsomega.2c04516 PMC9494440

[29] D. A. Bernards , D. J. Macaya , M. Nikolou , J. A. Defranco , S. Takamatsu , G. G. Malliaras , J. Mater. Chem. 2008, 18, 116.

[30] S. Han , S. Yamamoto , A. G. Polyravas , G. G. Malliaras , Adv. Mater. 2020, 32, 2004790. 10.1002/adma.202004790 33118196

[31] K. Guo , S. Wustoni , A. Koklu , E. Díaz‐Galicia , M. Moser , A. Hama , A. A. Alqahtani , A. N. Ahmad , F. S. Alhamlan , M. Shuaib , A. Pain , I. Mcculloch , S. T. Arold , R. Grünberg , S. Inal , Nat. Biomed. Eng. 2021, 5, 666. 34031558 10.1038/s41551-021-00734-9

[32] R. B. Rashid , X. Ji , J. Rivnay , Biosens. Bioelectron. 2021, 190, 113461. 34197997 10.1016/j.bios.2021.113461

[33] A. Marks , S. Griggs , N. Gasparini , M. Moser , Adv. Mater. Interfaces 2022, 9, 2102039.

[34] H. Lee , Y. Won , J. H. Oh , J. Polym. Sci. 2022, 60, 348.

[35] B. D. Paulsen , S. Fabiano , J. Rivnay , Ann. Rev. Mater. Res. 2021, 51, 73.

[36] R. Noriega , J. Rivnay , K. Vandewal , F. P. V. Koch , N. Stingelin , P. Smith , M. F. Toney , A. Salleo , Nat. Mater. 2013, 12, 1038. 23913173 10.1038/nmat3722

[37] J. Lee , A.‐R. Han , H. Yu , T. J. Shin , C. Yang , J. H. Oh , J. Am. Chem. Soc. 2013, 135, 9540. 23711152 10.1021/ja403949g

[38] S.‐H. Kang , D. Lee , W. Choi , J. H. Oh , C. Yang , Macromolecules 2022, 55, 4367.

[39] J. Y. Back , H. Yu , I. Song , I. Kang , H. Ahn , T. J. Shin , S.‐K. Kwon , J. H. Oh , Y.‐H. Kim , Chem. Mater. 2015, 27, 1732.

[40] K. K. Y. Lim , W. T. W. Takashima , T. E. T. Endo , M. R. M. Rikukawa , Jpn. J. Appl. Phys. 2000, 39, L872.

[41] S. Savagatrup , A. D. Printz , H. Wu , K. M. Rajan , E. J. Sawyer , A. V. Zaretski , C. J. Bettinger , D. J. Lipomi , Synth. Met. 2015, 203, 208.

[42] V. Ho , B. W. Boudouris , R. A. Segalman , Macromolecules 2010, 43, 7895.

[43] I. Mcculloch , M. Heeney , C. Bailey , K. Genevicius , I. Macdonald , M. Shkunov , D. Sparrowe , S. Tierney , R. Wagner , W. Zhang , M. L. Chabinyc , R. J. Kline , M. D. Mcgehee , M. F. Toney , Nat. Mater. 2006, 5, 328. 16547518 10.1038/nmat1612

[44] D.‐H. Lim , S.‐Y. Jang , M. Kang , S. Lee , Y.‐A. Kim , Y.‐J. Heo , M.‐H. Lee , D.‐Y. Kim , J. Mater. Chem. C 2017, 5, 10126.

[45] Y. Li , W. K. Tatum , J. W. Onorato , Y. Zhang , C. K. Luscombe , Macromolecules 2018, 51, 6352.

[46] Y. Li , W. K. Tatum , J. W. Onorato , S. D. Barajas , Y. Y. Yang , C. K. Luscombe , Polym. Chem. 2017, 8, 5185.

[47] H. Sirringhaus , Adv. Mater. 2014, 26, 1319. 24443057 10.1002/adma.201304346 PMC4515091

[48] S. Kim , D. Lee , J. Lee , Y. Cho , S.‐H. Kang , W. Choi , J. H. Oh , C. Yang , Chem. Mater. 2021, 33, 7499.

[49] S.‐H. Kang , A. Jeong , H. R. Lee , J. H. Oh , C. Yang , Chem. Mater. 2019, 31, 3831.

[50] A. Salleo , M. L. Chabinyc , M. S. Yang , R. A. Street , Appl. Phys. Lett. 2002, 81, 4383.

[51] R. J. Kline , M. D. Mcgehee , M. F. Toney , Nat. Mater. 2006, 5, 222.

[52] J.‐S. Kim , J.‐H. Kim , W. Lee , H. Yu , H. J. Kim , I. Song , M. Shin , J. H. Oh , U. Jeong , T.‐S. Kim , B. J. Kim , Macromolecules 2015, 48, 4339.

[53] R. Mauer , M. Kastler , F. Laquai , Adv. Funct. Mater. 2010, 20, 2085.

[54] A. R. Chew , R. Ghosh , V. Pakhnyuk , J. Onorato , E. C. Davidson , R. A. Segalman , C. K. Luscombe , F. C. Spano , A. Salleo , Adv. Funct. Mater. 2018, 28, 1804142.

[55] M. L. Tietze , L. Burtone , M. Riede , B. Lüssem , K. Leo , Phys. Rev. B 2012, 86, 035320.

[56] S. Olthof , S. Singh , S. K. Mohapatra , S. Barlow , S. R. Marder , B. Kippelen , A. Kahn , Appl. Phys. Lett. 2012, 101, 253303.

[57] S. Olthof , S. Mehraeen , S. K. Mohapatra , S. Barlow , V. Coropceanu , J.‐L. Brédas , S. R. Marder , A. Kahn , Phys. Rev. Lett. 2012, 109, 176601. 23215211 10.1103/PhysRevLett.109.176601

[58] S. Wang , M. Ha , M. Manno , C. D. Frisbie , C. Leighton , Nat. Commun. 2012, 3, 1210. 23169051 10.1038/ncomms2213

[59] B. D. Paulsen , C. D. Frisbie , J. Phys. Chem. C 2012, 116, 3132.

[60] A. Facchetti , Mater. Today 2007, 10, 28.

[61] M. Gsänger , D. Bialas , L. Huang , M. Stolte , F. Würthner , Adv. Mater. 2016, 28, 3615. 27028553 10.1002/adma.201505440

[62] A. F. Paterson , S. Singh , K. J. Fallon , T. Hodsden , Y. Han , B. C. Schroeder , H. Bronstein , M. Heeney , I. Mcculloch , T. D. Anthopoulos , Adv. Mater. 2018, 30, 1801079. 10.1002/adma.201801079 30022536

[63] C. J. Kousseff , R. Halaksa , Z. S. Parr , C. B. Nielsen , Chem. Rev. 2022, 122, 4397. 34491034 10.1021/acs.chemrev.1c00314

[64] J. Gao , C. Wang , D.‐W. Han , D.‐M. Shin , Chem. Sci. 2021, 12, 13248. 34777744 10.1039/d1sc04023e PMC8528010

[65] P. Colomban , A. Novak , J. Mol. Struct. 1988, 177, 277.

[66] R. G. Linford , S. Hackwood , Chem. Rev. 1981, 81, 327.

[67] N. Kamaya , K. Homma , Y. Yamakawa , M. Hirayama , R. Kanno , M. Yonemura , T. Kamiyama , Y. Kato , S. Hama , K. Kawamoto , A. Mitsui , Nat. Mater. 2011, 10, 682. 21804556 10.1038/nmat3066

[68] M. Petrowsky , R. Frech , J. Phys. Chem. B 2009, 113, 5996. 19338318 10.1021/jp810095g

[69] K. M. Diederichsen , H. G. Buss , B. D. Mccloskey , Macromolecules 2017, 50, 3831.

[70] G. Y. Gu , S. Bouvier , C. Wu , R. Laura , M. Rzeznik , K. M. Abraham , Electrochim. Acta 2000, 45, 3127.

[71] J. Park , A. Staiger , S. Mecking , K. I. Winey , ACS Macro Lett. 2022, 11, 1008. 35876880 10.1021/acsmacrolett.2c00288

[72] K. Ueno , J. Murai , K. Ikeda , S. Tsuzuki , M. Tsuchiya , R. Tatara , T. Mandai , Y. Umebayashi , K. Dokko , M. Watanabe , J. Phys. Chem. C 2016, 120, 15792.

[73] T. Miyake , M. Rolandi , J. Phys.: Condens. Matter 2016, 28, 023001. 26657711 10.1088/0953-8984/28/2/023001

[74] A. Melianas , T. J. Quill , G. Lecroy , Y. Tuchman , H. V. Loo , S. T. Keene , A. Giovannitti , H. R. Lee , I. P. Maria , I. Mcculloch , A. Salleo , Sci. Adv. 6, eabb2958. 10.1126/sciadv.abb2958 PMC7458436 32937458

[75] J. Liu , N. R. Davis , D. S. Liu , P. T. Hammond , J. Mater. Chem. 2012, 22, 15534.

[76] E. Stavrinidou , P. Leleux , H. Rajaona , D. Khodagholy , J. Rivnay , M. Lindau , S. Sanaur , G. G. Malliaras , Adv. Mater. 2013, 25, 4488. 23784809 10.1002/adma.201301240

[77] P. P Kumar , S. Yashonath , J. Chem. Sci. 2006, 118, 135.

[78] D. B. Shah , K. R. Olson , A. Karny , S. J. Mecham , J. M. Desimone , N. P. Balsara , J. Electrochem. Soc. 2017, 164, A3511.

[79] N. S. Schauser , R. Seshadri , R. A. Segalman , Mol. Syst. Des. Eng. 2019, 4, 263.

[80] E. Stavrinidou , O. Winther‐Jensen , B. S. Shekibi , V. Armel , J. Rivnay , E. Ismailova , S. Sanaur , G. G. Malliaras , B. Winther‐Jensen , Phys. Chem. Chem. Phys. 2014, 16, 2275. 24352071 10.1039/c3cp54061h

[81] D. G. Harman , R. Gorkin , L. Stevens , B. Thompson , K. Wagner , B. Weng , J. H. Y. Chung , M. In Het Panhuis , G. G. Wallace , Acta Biomater. 2015, 14, 33. 25484333 10.1016/j.actbio.2014.11.049

[82] X. Cui , J. F. Hetke , J. A. Wiler , D. J. Anderson , D. C. Martin , Sens. Actuator A Phys. 2001, 93, 8.

[83] H. Erothu , J. Kolomanska , P. Johnston , S. Schumann , D. Deribew , D. T. W. Toolan , A. Gregori , C. Dagron‐Lartigau , G. Portale , W. Bras , T. Arnold , A. Distler , R. C. Hiorns , P. Mokarian‐Tabari , T. W. Collins , J. R. Howse , P. D. Topham , Macromolecules 2015, 48, 2107.

[84] A. Gutacker , S. Adamczyk , A. Helfer , L. E. Garner , R. C. Evans , S. M. Fonseca , M. Knaapila , G. C. Bazan , H. D. Burrows , U. Scherf , J. Mater. Chem. 2010, 20, 1423.

[85] S. N. Patel , A. E. Javier , K. M. Beers , J. A. Pople , V. Ho , R. A. Segalman , N. P. Balsara , Nano Lett. 2012, 12, 4901. 22839306 10.1021/nl302454c

[86] A. Giovannitti , C. B. Nielsen , D.‐T. Sbircea , S. Inal , M. Donahue , M. R. Niazi , D. A. Hanifi , A. Amassian , G. G. Malliaras , J. Rivnay , I. Mcculloch , Nat. Commun. 2016, 7, 13066. 27713414 10.1038/ncomms13066 PMC5059848

[87] L. R. Savagian , A. M. Österholm , J. F. Ponder , K. J. Barth , J. Rivnay , J. R. Reynolds , Adv. Mater. 2018, 30, 1804647. 10.1002/adma.201804647 30368946

[88] A. Giovannitti , D.‐T. Sbircea , S. Inal , C. B. Nielsen , E. Bandiello , D. A. Hanifi , M. Sessolo , G. G. Malliaras , I. Mcculloch , J. Rivnay , Proc. Natl. Acad. Sci. USA 2016, 113, 12017. 27790983 10.1073/pnas.1608780113 PMC5087003

[89] S. Inal , J. Rivnay , P. Leleux , M. Ferro , M. Ramuz , J. C. Brendel , M. M. Schmidt , M. Thelakkat , G. G. Malliaras , Adv. Mater. 2014, 26, 7450. 25312252 10.1002/adma.201403150

[90] R. H. Karlsson , A. Herland , M. Hamedi , J. A. Wigenius , A. Åslund , X. Liu , M. Fahlman , O. Inganäs , P. Konradsson , Chem. Mater. 2009, 21, 1815.

[91] Q. Xie , S. Kuwabata , H. Yoneyama , J. Electroanal. Chem. 1997, 420, 219.

[92] J. Prokes , M. Varga , M. Vrnata , S. Valtera , J. Stejskal , D. Kopecký , Eur. Polym. J. 2019, 115, 290.

[93] Z. A. Lamport , H. F. Haneef , S. Anand , M. Waldrip , O. D. Jurchescu , J. Appl. Phys. 2018, 124, 071101.

[94] J. Rivnay , S. Inal , A. Salleo , R. M. Owens , M. Berggren , G. G. Malliaras , Nat. Rev. Mater. 2018, 3, 17086.

[95] D. Ohayon , V. Druet , S. Inal , Chem. Soc. Rev. 2023, 52, 1001. 36637165 10.1039/d2cs00920j

[96] H. Shen , A. Abtahi , B. Lussem , B. W. Boudouris , J. Mei , ACS Appl. Electron. Mater. 2021, 3, 2434.

[97] Y. Niu , Z. Qin , Y. Zhang , C. Chen , S. Liu , H. Chen , Mater. Futures 2023, 2, 042401.

[98] D. Khodagholy , J. Rivnay , M. Sessolo , M. Gurfinkel , P. Leleux , L. H. Jimison , E. Stavrinidou , T. Herve , S. Sanaur , R. M. Owens , G. G. Malliaras , Nat. Commun. 2013, 4, 2133. 23851620 10.1038/ncomms3133 PMC3717497

[99] S. G. Bucella , A. Luzio , E. Gann , L. Thomsen , C. R. Mcneill , G. Pace , A. Perinot , Z. Chen , A. Facchetti , M. Caironi , Nat. Commun. 2015, 6, 8394. 26403619 10.1038/ncomms9394 PMC4596007

[100] D. A. Bernards , G. G. Malliaras , Adv. Funct. Mater. 2007, 17, 3538.

[101] D. A. Koutsouras , P. Gkoupidenis , C. Stolz , V. Subramanian , G. G. Malliaras , D. C. Martin , ChemElectroChem 2017, 4, 2321.

[102] J. Rivnay , P. Leleux , M. Ferro , M. Sessolo , A. Williamson , D A. Koutsouras , D. Khodagholy , M. Ramuz , X. Strakosas , R. M. Owens , C. Benar , J.‐M. Badier , C. Bernard , G. G. Malliaras , Sci. Adv. 2015, 1, e1400251. 26601178 10.1126/sciadv.1400251 PMC4640642

[103] J. T. Friedlein , M. J. Donahue , S. E. Shaheen , G. G. Malliaras , R. R. Mcleod , Adv. Mater. 2016, 28, 8398. 27457055 10.1002/adma.201602684

[104] D. Ohayon , A. Savva , W. Du , B. D. Paulsen , I. Uguz , R. S. Ashraf , J. Rivnay , I. Mcculloch , S. Inal , ACS Appl. Mater. Interfaces 2021, 13, 4253. 33439636 10.1021/acsami.0c18599

[105] G. C. Faria , D. T. Duong , A. Salleo , Org. Electron. 2017, 45, 215.

[106] I. V. Krasnikova , M. A. Pogosova , A. O. Sanin , K. J. Stevenson , Chem. Mater. 2020, 32, 2232.

[107] D. Sotta , J. Bernard , V. Sauvant‐Moynot , Prog. Org. Coat. 2010, 69, 207.

[108] X. Qian , N. Gu , Z. Cheng , X. Yang , E. Wang , S. Dong , J. Solid State Electrochem. 2001, 6, 8.

[109] G. Garcia‐Belmonte , J. Bisquert , G. S. Popkirov , Appl. Phys. Lett. 2003, 83, 2178.

[110] X. Ren , P. G. Pickup , J. Phys. Chem. 1993, 97, 5356.

[111] M. Sheliakina , A. B. Mostert , P. Meredith , Adv. Funct. Mater. 2018, 28, 1805514.

[112] J. Bisquert , F. Fabregat‐Santiago , I. Mora‐Seró , G. Garcia‐Belmonte , E. M. Barea , E. Palomares , Inorg. Chim. Acta 2008, 361, 684.

[113] A. Elschner , W. Lövenich , MRS Bull. 2011, 36, 794.

[114] N. A. Shahrim , Z. Ahmad , A. Wong Azman , Y. Fachmi Buys , N. Sarifuddin , Mater. Adv. 2021, 2, 7118.

[115] I. Petsagkourakis , N. Kim , K. Tybrandt , I. Zozoulenko , X. Crispin , Adv. Electron. Mater. 2019, 5, 1800918.

[116] Y. Jiang , T. Liu , Y. Zhou , Adv. Funct. Mater. 2020, 30, 2006213.

[117] Y. H. Kim , C. Sachse , M. L. Machala , C. May , L. Müller‐Meskamp , K. Leo , Adv. Funct. Mater. 2011, 21, 1076.

[118] L. V. Lingstedt , M. Ghittorelli , H. Lu , D. A. Koutsouras , T. Marszalek , F. Torricelli , N. I Craciun , P. Gkoupidenis , P. W. M. Blom , Adv. Electron. Mater. 2019, 5, 1800804.

[119] D. Alemu , H.‐Y. Wei , K.‐C. Ho , C.‐W. Chu , Energy Environ. Sci. 2012, 5, 9662.

[120] A. De Izarra , S. Park , J. Lee , Y. Lansac , Y. H. Jang , J. Am. Chem. Soc. 2018, 140, 5375. 29633844 10.1021/jacs.7b10306

[121] Y. Xia , K. Sun , J. Ouyang , Energy Environ. Sci. 2012, 5, 5325.

[122] F. Wu , P. Li , K. Sun , Y. Zhou , W. Chen , J. Fu , M. Li , S. Lu , D. Wei , X. Tang , Z. Zang , L. Sun , X. Liu , J. Ouyang , Adv. Electron. Mater. 2017, 3, 1700047.

[123] M. N. Gueye , A. Carella , J. Faure‐Vincent , R. Demadrille , J.‐P. Simonato , Prog. Mater. Sci. 2020, 108, 100616.

[124] O. Bubnova , X. Crispin , Energy Environ. Sci. 2012, 5, 9345.

[125] W. Zhang , Z. Su , X. Zhang , W. Wang , Z. Li , View 2022, 3, 20220030.

[126] N. Massonnet , A. Carella , O. Jaudouin , P. Rannou , G. Laval , C. Celle , J.‐P. Simonato , J. Mater. Chem. C 2014, 2, 1278.

[127] P. Yadav , A. Patra , Polym. Chem. 2020, 11, 7275.

[128] D. R. Evans , Chem. Commun. 2022, 58, 4553. 10.1039/d2cc01100j 35332350

[129] D. Mantione , I. Del Agua , A. Sanchez‐Sanchez , D. Mecerreyes , Polymers 2017, 9, 354. 30971030 10.3390/polym9080354 PMC6418870

[130] H.‐E. Yin , C.‐F. Lee , W.‐Y. Chiu , Polymer 2011, 52, 5065.

[131] W. Cho , S. Im , S. Kim , S. Kim , J. Kim , Polymers 2016, 8, 189.30979282

[132] C. Pozo‐Gonzalo , R. Marcilla , M. Salsamendi , D. Mecerreyes , J. A. Pomposo , J. Rodríguez , H. J. Bolink , J. Polym. Sci. Part A: Polym. Chem. 2008, 46, 3150.

[133] Y. Li , N. Hong , J. Mater. Chem. A 2015, 3, 21537.

[134] Y. Li , M. Liu , Y. Li , K. Yuan , L. Xu , W. Yu , R. Chen , X. Qiu , H.‐L. Yip , Adv. Energy Mater. 2017, 7, 1601499.

[135] A. I. Hofmann , W. T. T. Smaal , M. Mumtaz , D. Katsigiannopoulos , C. Brochon , F. Schütze , O. R. Hild , E. Cloutet , G. Hadziioannou , Angew. Chem., Int. Ed. 2015, 54, 8506. 10.1002/anie.201503024 26033573

[136] J. F. Ponder , A. M. Österholm , J. R. Reynolds , Macromolecules 2016, 49, 2106.

[137] J. F. Ponder , B. Schmatz , J. L. Hernandez , J. R. Reynolds , J. Mater. Chem. C 2018, 6, 1064.

[138] S. Ertan , C. Kaynak , A. Cihaner , J. Polym. Sci. Part A: Polym. Chem. 2017, 55, 3935.

[139] E. W. Fager , J. Am. Chem. Soc. 1945, 67, 2217.

[140] D. Hu , B. Lu , X. Duan , J. Xu , L. Zhang , K. Zhang , S. Zhang , S. Zhen , RSC Adv. 2014, 4, 35597.

[141] L. K. Povlich , J. C. Cho , M. K. Leach , J. M. Corey , J. Kim , D. C. Martin , Biochim. Biophys. Acta Gen. Subj. 2013, 1830, 4288. 10.1016/j.bbagen.2012.10.017 23103748

[142] M. Horikawa , T. Fujiki , T. Shirosaki , N. Ryu , H. Sakurai , S. Nagaoka , H. Ihara , J. Mater. Chem. C 2015, 3, 8881.

[143] A. I. Hofmann , D. Katsigiannopoulos , M. Mumtaz , I. Petsagkourakis , G. Pecastaings , G. Fleury , C. Schatz , E. Pavlopoulou , C. Brochon , G. Hadziioannou , E. Cloutet , Macromolecules 2017, 50, 1959.

[144] I. Del Agua , D. Mantione , N. Casado , A. Sanchez‐Sanchez , G. G. Malliaras , D. Mecerreyes , ACS Macro Lett. 2017, 6, 473. 35610866 10.1021/acsmacrolett.7b00104

[145] S. Inal , G. G. Malliaras , J. Rivnay , Nat. Commun. 2017, 8, 1767. 29176599 10.1038/s41467-017-01812-w PMC5701155

[146] N. A. Kukhta , A. Marks , C. K. Luscombe , Chem. Rev. 2022, 122, 4325. 34902244 10.1021/acs.chemrev.1c00266 PMC8874907

[147] C. B. Nielsen , A. Giovannitti , D.‐T. Sbircea , E. Bandiello , M. R. Niazi , D. A. Hanifi , M. Sessolo , A. Amassian , G. G. Malliaras , J. Rivnay , I. Mcculloch , J. Am. Chem. Soc. 2016, 138, 10252. 27444189 10.1021/jacs.6b05280 PMC4991841

[148] Y. He , N. A. Kukhta , A. Marks , C. K. Luscombe , J. Mater. Chem. C 2022, 10, 2314. 10.1039/d1tc05229b PMC8852261 35310858

[149] M. Moser , T. C. Hidalgo , J. Surgailis , J. Gladisch , S. Ghosh , R. Sheelamanthula , Q. Thiburce , A. Giovannitti , A. Salleo , N. Gasparini , A. Wadsworth , I. Zozoulenko , M. Berggren , E. Stavrinidou , S. Inal , I. Mcculloch , Adv. Mater. 2020, 32, 2002748. 10.1002/adma.202002748 32754923

[150] R. K. Hallani , B. D. Paulsen , A. J. Petty , R. Sheelamanthula , M. Moser , K. J. Thorley , W. Sohn , R. B. Rashid , A. Savva , S. Moro , J. P. Parker , O. Drury , M. Alsufyani , M. Neophytou , J. Kosco , S. Inal , G. Costantini , J. Rivnay , I. Mcculloch , J. Am. Chem. Soc. 2021, 143, 11007. 34192463 10.1021/jacs.1c03516

[151] L. Lan , J. Chen , Y. Wang , P. Li , Y. Yu , G. Zhu , Z. Li , T. Lei , W. Yue , I. Mcculloch , Chem. Mater. 2022, 34, 1666.

[152] N. Siemons , D. Pearce , C. Cendra , H. Yu , S. M. Tuladhar , R. K. Hallani , R. Sheelamanthula , G. S. Lecroy , L. Siemons , A. J. P. White , I. Mcculloch , A. Salleo , J. M. Frost , A. Giovannitti , J. Nelson , Adv. Mater. 2022, 34, 2204258. 10.1002/adma.202204258 35946142

[153] X. Wu , Q. Liu , A. Surendran , S. E. Bottle , P. Sonar , W. L. Leong , Adv. Electron. Mater. 2021, 7, 2000701.

[154] M. Moser , A. Savva , K. Thorley , B. D. Paulsen , T. C. Hidalgo , D. Ohayon , H. Chen , A. Giovannitti , A. Marks , N. Gasparini , A. Wadsworth , J. Rivnay , S. Inal , I. Mcculloch , Angew. Chem., Int. Ed. 2021, 60, 7777. 10.1002/anie.202014078 33259685

[155] S. Cong , J. Chen , L. Wang , L. Lan , Y. Wang , H. Dai , H. Liao , Y. Zhou , Y. Yu , J. Duan , Z. Li , I. Mcculloch , W. Yue , Adv. Funct. Mater. 2022, 32, 2201821.

[156] D. Jeong , I.‐Y. Jo , S. Lee , J. H. Kim , Y. Kim , D. Kim , J. R. Reynolds , M.‐H. Yoon , B. J. Kim , Adv. Funct. Mater. 2022, 32, 2111950.

[157] J. Surgailis , A. Savva , V. Druet , B. D. Paulsen , R. Wu , A. Hamidi‐Sakr , D. Ohayon , G. Nikiforidis , X. Chen , I. Mcculloch , J. Rivnay , S. Inal , Adv. Funct. Mater. 2021, 31, 2010165.

[158] A. Marks , X. Chen , R. Wu , R. B. Rashid , W. Jin , B. D. Paulsen , M. Moser , X. Ji , S. Griggs , D. Meli , X. Wu , H. Bristow , J. Strzalka , N. Gasparini , G. Costantini , S. Fabiano , J. Rivnay , I. Mcculloch , J. Am. Chem. Soc. 2022, 144, 4642. 35257589 10.1021/jacs.2c00735 PMC9084553

[159] P. Li , J. Shi , Y. Lei , Z. Huang , T. Lei , Nat. Commun. 2022, 13, 5970. 36216813 10.1038/s41467-022-33553-w PMC9551099

[160] A. Koklu , S. Wustoni , K. Guo , R. Silva , L. Salvigni , A. Hama , E. Diaz‐Galicia , M. Moser , A. Marks , I. Mcculloch , R. Grünberg , S. T. Arold , S. Inal , Adv. Mater. 2022, 34, 2202972. 10.1002/adma.202202972 35772173

[161] Y. Song , H. Zhang , T. Mukhopadhyaya , A. S. Hall , H. E. Katz , Biosens. Bioelectron. 2022, 216, 114691. 36113388 10.1016/j.bios.2022.114691

[162] J. Fan , S. Parr , S. Kang , M. Gupta , Nanoscale 2023, 15, 5476. 36852643 10.1039/d2nr06485e

[163] H. Liu , A. Yang , J. Song , N. Wang , P. Lam , Y. Li , H. K.‐W. Law , F. Yan , Sci. Adv. 2021, 7, eabg8387. 34524851 10.1126/sciadv.abg8387 PMC8443172

[164] W. Li , J. Jin , T. Xiong , P. Yu , L. Mao , Angew. Chem., Int. Ed. 2022, 61, e202204134. 10.1002/anie.202204134 35583258

[165] K. Xie , N. Wang , X. Lin , Z. Wang , X. Zhao , P. Fang , H. Yue , J. Kim , J. Luo , S. Cui , F. Yan , P. Shi , eLife 2020, 9, e50345. 32043970 10.7554/eLife.50345 PMC7075691

[166] L. M. M. Ferro , L. Merces , D. H. S. De Camargo , C. C. Bof Bufon , Adv. Mater. 2021, 33, 2101518. 10.1002/adma.202101518 34061409

[167] B. Burtscher , P. A. Manco Urbina , C. Diacci , S. Borghi , M. Pinti , A. Cossarizza , C. Salvarani , M. Berggren , F. Biscarini , D. T. Simon , C. A. Bortolotti , Adv. Healthcare Mater. 2021, 10, 2100955. 10.1002/adhm.202100955 PMC11469060 34423579

[168] R. Ban , M.‐J. Lu , J. Hu , C.‐J. Li , Y.‐M. Li , G. Gao , C.‐S. Wang , F.‐Y. Kong , H. Zhou , P. Lin , W.‐W. Zhao , Biosens. Bioelectron. 2022, 218, 114752. 36240627 10.1016/j.bios.2022.114752

[169] Z. Shi , Z. Xu , J. Hu , W. Wei , X. Zeng , W.‐W. Zhao , P. Lin , Biosens. Bioelectron. 2022, 201, 113958. 34996003 10.1016/j.bios.2021.113958

[170] X. Guo , Q. Cao , Y. Liu , T. He , J. Liu , Si Huang , H. Tang , M. Ma , Anal. Chem. 2020, 92, 908. 31769281 10.1021/acs.analchem.9b03718

[171] A. M. Pappa , D. Ohayon , A. Giovannitti , I. P. Maria , A. Savva , I. Uguz , J. Rivnay , I. Mcculloch , R. M. Owens , S. Inal , Sci. Adv. 2018, 4, eaat0911. 29942860 10.1126/sciadv.aat0911 PMC6014717

[172] F. Gentile , F. Vurro , M. Janni , R. Manfredi , F. Cellini , A. Petrozza , A. Zappettini , N. Coppedè , Adv. Electron. Mater. 2022, 8, 2200092.

[173] D. A. Koutsouras , K. Lieberth , F. Torricelli , P. Gkoupidenis , P. W. M. Blom , Adv. Mater. Technol. 2021, 6, 2100591. 10.1002/adhm.202100845 PMC11468701 34309226

[174] J. N. Arthur , S. Burns , C. M. Cole , Q. T. Barthelme , S. D. Yambem , J. Mater. Chem. C 2022, 10, 13964.

[175] P. Romele , P. Gkoupidenis , D. A. Koutsouras , K. Lieberth , Z. M. Kovács‐Vajna , P. W. M. Blom , F. Torricelli , Nat. Commun. 2020, 11, 3743. 32719350 10.1038/s41467-020-17547-0 PMC7385487

[176] J. Wu , H. Liu , W. Chen , B. Ma , H. Ju , Nat. Rev. Bioeng. 2023, 1, 346. 37168735 10.1038/s44222-023-00032-w PMC9951169

[177] L. Bai , C. G. Elósegui , W. Li , P. Yu , J. Fei , L. Mao , Front. Chem. 2019, 7, 00313. 10.3389/fchem.2019.00313 PMC6514146 31134185

[178] Z. Zhang , P. Ma , R. Ahmed , J. Wang , D. Akin , F. Soto , Bi‐F Liu , P. Li , U. Demirci , Adv. Mater. 2022, 34, 2103646. 10.1002/adma.202103646 34623709

[179] S. D. Niyonambaza , P. Kumar , P. Xing , J. Mathault , P. De Koninck , E. Boisselier , M. Boukadoum , A. Miled , Appl. Sci. 2019, 9, 4719.

[180] R. Strack , Nat. Methods 2019, 16, 17. 10.1038/s41592-018-0267-9 30573839

[181] S. Chen , A. Surendran , X. Wu , W. L. Leong , Adv. Funct. Mater. 2020, 30, 2006186.

[182] X. Wang , X. Meng , Y. Zhu , H. Ling , Y. Chen , Z. Li , M. C. Hartel , M. R. Dokmeci , S. Zhang , A. Khademhosseini , IEEE Electron Device Lett. 2021, 42, 46. 33746352 10.1109/led.2020.3042310 PMC7978230

[183] G. D. Tabi , J. S. Kim , B. Nketia‐Yawson , D. H. Kim , Y.‐Y. Noh , J. Mater. Chem. C 2020, 8, 17107.

[184] M. Zhang , Z. Tang , X. Liu , J. Van Der Spiegel , Nat. Electron. 2020, 3, 191.

[185] D. O. Adewole , M. D. Serruya , J. P. Harris , J. C. Burrell , D. Petrov , H. I. Chen , J. A. Wolf , D. K. Cullen , 2016, 44, 123. 10.1615/CritRevBiomedEng.2016017198 PMC5541680 27652455

[186] B. P. Bean , Nat. Rev. Neurosci. 2007, 8, 451. 17514198 10.1038/nrn2148

[187] P. Fromherz , ChemPhysChem 2002, 3, 276. 12503174 10.1002/1439-7641(20020315)3:3<276::AID-CPHC276>3.0.CO;2-A

[188] B. Yao , Y. Yan , Q. Cui , S. Duan , C. Wang , Y. Du , Y. Zhao , D. Wu , S. Wu , X. Zhu , T. Hsiai , X. He , Matter 2022, 5, 4407.

[189] D. Khodagholy , J. N. Gelinas , T. Thesen , W. Doyle , O. Devinsky , G. G. Malliaras , G. Buzsáki , Nat. Neurosci. 2015, 18, 310. 25531570 10.1038/nn.3905 PMC4308485

[190] W. Lee , S. Kobayashi , M. Nagase , Y. Jimbo , I. Saito , Y. Inoue , T. Yambe , M. Sekino , G. G. Malliaras , T. Yokota , M. Tanaka , T. Someya , Sci. Adv. 2018, 4, eaau2426. 30345362 10.1126/sciadv.aau2426 PMC6195340

[191] D. O. Adewole , L. A. Struzyna , J. C. Burrell , J. P. Harris , A. D. Nemes , D. Petrov , R. H. Kraft , H. I. Chen , M. D. Serruya , J. A. Wolf , D. K Cullen , Sci. Adv. 2021, 7, eaay5347. 33523957 10.1126/sciadv.aay5347 PMC10670819

[192] M. Bianchi , A. De Salvo , M. Asplund , S. Carli , M. Di Lauro , A. Schulze‐Bonhage , T. Stieglitz , L. Fadiga , F. Biscarini , Adv. Sci. 2022, 9, 2104701. 10.1002/advs.202104701 PMC9036021 35191224

[193] S. Vassanelli , M. Mahmud , Front. Neurosci. 2016, 10, 00062. 10.3389/fnins.2016.00248 PMC4889584 27313507

[194] S. T. Keene , C. Lubrano , S. Kazemzadeh , A. Melianas , Y. Tuchman , G. Polino , P. Scognamiglio , L. Cinà , A. Salleo , Y. Van De Burgt , F. Santoro , Nat. Mater. 2020, 19, 969. 32541935 10.1038/s41563-020-0703-y

[195] A. Jonsson , Z. Song , D. Nilsson , B. A. Meyerson , D. T. Simon , B. Linderoth , M. Berggren , Sci. Adv. 2015, 1, e1500039. 26601181 10.1126/sciadv.1500039 PMC4640645

[196] J. Liu , X. Zhang , Y. Liu , M. Rodrigo , P. D. Loftus , J. Aparicio‐Valenzuela , J. Zheng , T. Pong , K. J. Cyr , M. Babakhanian , J. Hasi , J. Li , Y. Jiang , C. J. Kenney , P. J. Wang , A. M. Lee , Z. Bao , Proc. Natl. Acad. Sci. USA 2020, 117, 14769. 32541030 10.1073/pnas.2000207117 PMC7334471

[197] Y. Zhang , E R. W. Van Doremaele , G. Ye , T. Stevens , J. Song , R. C. Chiechi , Y. Van De Burgt , Adv. Mater. 2022, 34, 2200393. 10.1002/adma.202200393 35334499

[198] P. Gkoupidenis , N. Schaefer , B. Garlan , G. G. Malliaras , Adv. Mater. 2015, 27, 7176. 26456708 10.1002/adma.201503674

[199] W. Xu , S.‐Y. Min , H. Hwang , T.‐W. Lee , Sci. Adv. 2016, 2, e1501326. 27386556 10.1126/sciadv.1501326 PMC4928881

[200] H. R. Lee , D. Lee , J. H. Oh , Adv. Mater. 2021, 33, 2100119. 10.1002/adma.202100119 33754389

[201] G.‐T. Go , Y. Lee , D.‐G. Seo , T.‐W. Lee , Adv. Mater. 2022, 34, 2201864. 10.1002/adma.202201864 35925610

[202] Y. Choi , S. Oh , C. Qian , J.‐H. Park , J. H. Cho , Nat. Commun. 2020, 11, 4595. 32929064 10.1038/s41467-020-17850-w PMC7490352

[203] E. Zeglio , O. Inganäs , Adv. Mater. 2018, 30, 1800941. 10.1002/adma.201800941 30022545

[204] W. Huang , J. Chen , Y. Yao , D. Zheng , X. Ji , L.‐W. Feng , D. Moore , N. R. Glavin , M. Xie , Y. Chen , R. M. Pankow , A. Surendran , Z. Wang , Y. Xia , L. Bai , J. Rivnay , J. Ping , X. Guo , Y. Cheng , T. J. Marks , A. Facchetti , Nature 2023, 613, 496. 36653571 10.1038/s41586-022-05592-2 PMC9849123

[205] A. Weissbach , L. M. Bongartz , M. Cucchi , H. Tseng , K. Leo , H. Kleemann , J. Mater. Chem. C 2022, 10, 2656. 10.1038/s41467-022-32182-7 PMC9349225 35922437

[206] P. C. Harikesh , C.‐Y. Yang , H.‐Y. Wu , S. Zhang , M. J. Donahue , A. S. Caravaca , J.‐D. Huang , P. S. Olofsson , M. Berggren , D. Tu , S. Fabiano , Nat. Mater. 2023, 22, 242. 36635590 10.1038/s41563-022-01450-8 PMC9894750

[207] S. E. Chen , R. Giridharagopal , D. S. Ginger , Nat. Mater. 2023, 22, 416. 37002500 10.1038/s41563-023-01509-0

[208] P. C. Harikesh , C.‐Y. Yang , D. Tu , J. Y. Gerasimov , A. M. Dar , A. Armada‐Moreira , M. Massetti , R. Kroon , D. Bliman , R. Olsson , E. Stavrinidou , M. Berggren , S. Fabiano , Nat. Commun. 2022, 13, 901. 35194026 10.1038/s41467-022-28483-6 PMC8863887

[209] Y. Kim , A. Chortos , W. Xu , Y. Liu , J. Y. Oh , D. Son , J. Kang , A. M. Foudeh , C. Zhu , Y. Lee , S. Niu , J. Liu , R. Pfattner , Z. Bao , T.‐W. Lee , Science 2018, 360, 998. 29853682 10.1126/science.aao0098

[210] J. F. Otto , Y. Yang , W. N. Frankel , H. S White , K. S. Wilcox , J. Neurosci. 2006, 26, 2053. 16481438 10.1523/JNEUROSCI.1575-05.2006 PMC6674924

[211] M. A. Ungless , X. Gasull , E. T. Walters , J. Neurophysiol. 2002, 87, 2408. 11976378 10.1152/jn.2002.87.5.2408

[212] A. L. Hodgkin , A. F. Huxley , J. Physiol. 1952, 117, 500. 12991237 10.1113/jphysiol.1952.sp004764 PMC1392413

[213] E. M. Izhikevich , IEEE Trans. Neural Netw. 2003, 14, 1569. 18244602 10.1109/TNN.2003.820440

[214] D.‐G. Seo , Y. Lee , G.‐T. Go , M. Pei , S. Jung , Y. H. Jeong , W. Lee , H.‐L. Park , S.‐W. Kim , H. Yang , C. Yang , T.‐W. Lee , Nano Energy 2019, 65, 104035.

[215] Y. Lee , J. Y. Oh , W. Xu , O. Kim , T. R. Kim , J. Kang , Y. Kim , D. Son , J. B.‐H. Tok , M. J. Park , Z. Bao , T.‐W. Lee , Sci. Adv. 2018, 4, eaat7387. 30480091 10.1126/sciadv.aat7387 PMC6251720

[216] Y. Lee , Y. Liu , D.‐G. Seo , J. Y. Oh , Y. Kim , J. Li , J. Kang , J. Kim , J. Mun , A. M. Foudeh , Z. Bao , T.‐W. Lee , Nat. Biomed. Eng. 2023, 7, 511. 35970931 10.1038/s41551-022-00918-x

[217] S. T. M. Tan , A. Gumyusenge , T. J. Quill , G. S. Lecroy , G. E. Bonacchini , I. Denti , A. Salleo , Adv. Mater. 2022, 34, 2110406. 10.1002/adma.202110406 35434865

[218] W. Cheng , M. Moreno‐Gonzalez , K. Hu , C. Krzyszkowski , D. J. Dvorak , D. M. Weekes , B. Tam , C. P. Berlinguette , iScience 2018, 10, 80. 30508720 10.1016/j.isci.2018.11.014 PMC6277218

[219] D. Moia , A. Giovannitti , A. A. Szumska , I. P. Maria , E. Rezasoltani , M. Sachs , M. Schnurr , P. R. F. Barnes , I. Mcculloch , J. Nelson , Energy Environ. Sci. 2019, 12, 1349.

[220] S. Fabiano , S. Braun , X. Liu , E. Weverberghs , P. Gerbaux , M. Fahlman , M. Berggren , X. Crispin , Adv. Mater. 2014, 26, 6000. 25043202 10.1002/adma.201401986

[221] X. Li , K. Perera , J. He , A. Gumyusenge , J. Mei , J. Mater. Chem. C 2019, 7, 12761.

[222] D. E. Shen , A. M. Österholm , J. R. Reynolds , J. Mater. Chem. C 2015, 3, 9715.

[223] J. Kim , M. Rémond , D. Kim , H. Jang , E. Kim , Adv. Mater. Technol. 2020, 5, 1900890.

[224] W. Li , T. Bai , G. Fu , Q. Zhang , J. Liu , H. Wang , Y. Sun , H. Yan , Sol. Energy Mater. Sol. Cells 2022, 240, 111709.

[225] T. A. Welsh , E. R. Draper , RSC Adv. 2021, 11, 5245. 35424438 10.1039/d0ra10346b PMC8694694

[226] R. J. Mortimer , Ann. Rev. Mater. Res. 2011, 41, 241.

[227] J. Rivnay , R. M. Owens , G. G. Malliaras , Chem. Mater. 2014, 26, 679.

[228] M. A. Farahmand Nejad , S. Ranjbar , C. Parolo , E P. Nguyen , R. Álvarez‐Diduk , M. R. Hormozi‐Nezhad , A. Merkoçi , Mater. Today 2021, 50, 476.

[229] Z. Wang , X. Wang , S. Cong , F. Geng , Z. Zhao , Mater. Sci. Eng. R Rep. 2020, 140, 100524.

[230] H.‐H. Chou , A. Nguyen , A. Chortos , J. W. F. To , C. Lu , J. Mei , T. Kurosawa , W.‐G. Bae , J. B.‐H. Tok , Z. Bao , Nat. Commun. 2015, 6, 8011. 26300307 10.1038/ncomms9011 PMC4560774

[231] Z. Yu , G. Cai , X. Liu , D. Tang , Anal. Chem. 2021, 93, 2916. 33492928 10.1021/acs.analchem.0c04501

[232] J. Koo , V. Amoli , S. Y. Kim , C. Lee , J. Kim , S.‐M. Park , J. Kim , J. M. Ahn , K. J. Jung , D. H. Kim , Nano Energy 2020, 78, 105199.

